# The effect of trusting contexts in social dilemmas with collective and individual solutions

**DOI:** 10.1038/s41598-024-77190-3

**Published:** 2024-10-30

**Authors:** Sergio Lo Iacono, Burak Sonmez, Malcolm Fairbrother

**Affiliations:** 1 University of Essex, Colchester, UK; 2 University College London, London, UK; 3 Uppsala Universitet, Uppsala, Sweden; 4 Institute for Future Studies, Stockholm, Sweden

**Keywords:** Climate-change mitigation, Psychology and behaviour

## Abstract

Trust encourages members of communities to cooperate and provide public goods. However, the literature has yet to fully investigate how high and low trusting communities deal with collective action dilemmas with multiple solutions. The latter may raise the risk of coordination failure. Using a preregistered interactive experiment (N participants/groups = 371/70), we investigated people’s decisions when they have three possible choices in confronting a collective action dilemma: investing in an individual solution, investing in a collective solution, and free-riding. We manipulated the incentives for trusting and trustworthy interactions among community members, and, consistent with our expectations, we found that people in high-trust contexts invest more in collective solutions, compared to people in low-trust contexts. In the latter case, participants opted more for individual solutions, using resources less efficiently. However, we found no difference in the prevalence of free-riding in high- compared to low-trust contexts.

**Supplementary Information:**

The online version contains supplementary material available at 10.1038/s41598-024-77190-3.

## Introduction

Experimental and observational research has identified a variety of factors influencing the chances of groups overcoming collective action dilemmas. Such factors include community wealth, inequality, economic growth, and mean levels of individual institutional and social trust^1–11^. Especially given that serious problems of environmental degradation (e.g., climate change) can be understood and formalized as collective action dilemmas, we urgently need to better understand what makes the successful resolution of such dilemmas more or less likely. Polluters externalize their costs and engage in actions that are more beneficial than harmful for themselves but more harmful than beneficial for society as a whole^12^. Conversely, the mitigation of environmental harms benefits whole communities, including members who do not contribute to that mitigation. In the absence of adequate monitoring and enforcement, therefore, individuals can be tempted to free-ride^13–17^. Under what conditions, then, do individuals resist the temptation to free-ride, and, as Laffont (p. 431) classically puts it, “not leave their beer cans on beaches?”^18^.

A variety of experimental studies have investigated individuals’ decision-making processes with respect to social dilemmas and the mitigation of environmental degradation, which is typically formalized in the literature as a public goods game with threshold^19–21^. Yet, such studies have not considered the challenge of public goods provision when individuals can, instead of investing only in collective solutions, opt to invest in individual solutions to some shared problem. The availability of an individual solution may reduce the incentives for participants to cooperate on a more efficient collective solution—even if the individual solution addresses the free-rider problem^22–24^.

To illustrate this challenge, consider the example of a community of farmers who need their crops to be pollinated. As an individual solution, farmers may opt to pay workers to hand-pollinate their crops, or pay someone to place beehives on their land temporarily. Alternatively, as a collective solution, the farmers might agree that everyone will make an effort to protect local pollinators, such as by limiting the use of certain pesticides, or letting some amount of their land go wild, so there is some appropriate pollinator habitat. Third, however, a given farmer might decide not to invest in any pollination services, but merely hope that other farmers do. Even if not all farmers invest, potentially enough will, such that all farmers can get their crops pollinated. The first, individual, solution costs the farmer, and is expensive, but is better than no pollination at all. The second, collective, solution is also individually costly, but more cost-effective than the individual solution—if the solution is achieved. The third option, free-riding, costs the individual nothing; but if too many farmers make this choice, the outcome is negative for all. A similar logic can be applied to a variety of other social dilemmas where shared problems generated by environmental degradation can be addressed via both individual and collective solutions. For instance, a commuter using a fossil fuel-powered private vehicle could seek to reduce their impact on the climate by switching to public transport (a collective solution), or by purchasing an electric vehicle (a private solution). They might also simply hope that the efforts of others will be enough to reduce greenhouse gas emissions to a sustainable level, and stay with their current daily (high-polluting) form of commute (free-riding).

The freedom of individuals to pursue individual or collective solutions and the different levels of efficiency of each solution point out new layers of complexity of such environmental social dilemmas, which imply a rich set of risks that communities face when dealing with these issues: free-riding, coordination failure, and inefficiency^23^. Recent studies show that as self-reliance grows, groups become less effective in generating public goods, magnifying wealth disparities, eroding social unity, and accentuating divisions in approaches to governing shared resources^22,24^. Little is known, however, about how different trusting contexts can lead individuals to confront collective action problems with both individual and collective solutions, when there is no institution compelling people to contribute to the collective solution.

We conducted a pre-registered real-time experiment consisting of two interactive games followed by a short questionnaire gathering information on participants’ characteristics. In the first game, participants played a modified binary Trust Game (TG) in which we randomly assigned groups of participants to either of two possible sets of payoffs^4,25,26^. Participants in the first treatment group played the binary TG with payoffs that discouraged cooperative exchanges, while participants in the other group played with payoffs providing strong incentives for cooperative exchanges. Engaging in these two different contexts leads people in high-trust contexts not only to exhibit more frequent trusting and trustworthy behaviors compared to people in low-trust context, but also report more trusting and trustworthy attitudes toward other participants^4^. In the second game, participants played an Independence Dilemma^23^ in which they could choose to invest their resources in an individual pool, a shared public pool, or not to invest in either and keep the resources for themselves. By reaching a specific threshold in the individual or public pool, participants could keep the resources accumulated, otherwise these would be lost. Insights from signaling theory in sociology suggest that individuals use information acquired within a specific context to decide on whether to cooperate across various relevant situations^27,28^: what people learn about others in one context can work as a signal of cooperativeness, which will feed into their decision to coordinate in other, different social exchanges with the same individuals^4,27,29–33^. These results suggest that decision making in the second game will be likely affected by what participants observe or experience in terms of trusting and trustworthy behaviors in the first game. In particular, high-trust contexts seem to lead to a higher likelihood to achieve the public good in a one-shot setting with binary choice^4^. However, it is unclear whether this effect would persist when a continued, repeated effort is required to achieve the public good and multiple solutions are available to the individual, increasing the chances of coordination failure and inefficiency. In this sense, how high-trust contexts perform in the case of environmental social dilemmas may be more nuanced.

On the one hand, individuals in such contexts have reason to believe that others will act in a cooperative and responsible manner, and can reasonably expect that others will reciprocate their own cooperative behavior. Positive expectations of others may lead any given individual also to behave cooperatively. However, on the other hand, each individual stands to gain more from exploiting others’ contributions, by free-riding, insofar as others invest in a collective solution. High-trust contexts could therefore encourage greater investment in collective solutions, but also encourage more free-riding, compared to low-trust contexts. In the latter, as others can be expected not to contribute much, there is little to free-ride on and there should be a stronger preference for the individual solution.

Some of the most notable collective action problems in the world today include environmental problems, in which actors (whether individuals or firms) impose the costs of their actions onto others. It is because others pay the costs of each polluter’s actions that polluters can make choices that are more costly than beneficial for society as a whole. At the same time, actors do not always impose costs on others when they could. Many people do not litter, for example, and engage in an effort to recycle—even at some personal inconvenience. The social sciences have long grappled with the challenge of identifying conditions under which either of these outcomes—free-riding or cooperation—is more likely than the other.

We know, for example, that cooperative outcomes can more easily be achieved in contexts where participants know and can monitor each other, and can punish any rule-breaking^13–16,34^. Past research has shown that individuals are more likely to cooperate if they believe others will do so – e.g., on the basis of shared empirical and normative expectations^35–39^. In that vein, we expect that if people experience a high-trust context—i.e., learn that others in their social environment are generally cooperative—they will be more likely to believe that the majority of people will contribute to the public good. This should, in turn, increase the chances of individuals choosing to invest in the collective solution. On the other hand, if people experience a low-trust context—i.e., learn that others are generally uncooperative—we expect they will invest more in the individual solution:

### H1a

*People who experience a high-trust context will be more likely to opt for collective solutions than people who experience a low-trust context.*

### H1b

*People who experience a low-trust context will be more likely to opt for individual solutions than people who experience a high-trust context.*

Furthermore, however, since experiencing a high-trust context leads people to believe that others in the community will opt for the collective solution, the temptation to free-ride should be higher in the high-trust context than the low-trust context. Hence, we also expect the following:

### H2

*People who experience a high-trust context will be more likely to opt for free-riding behaviors than people who experienced a low-trust context.*

Despite the more frequent free-riding behaviors in the high-trust context, we expect that the chances of achieving collective solutions and a higher coordination efficiency (i.e., solving a shared problem by coordinating on the collective solution minimizing the waste of resources) will still be higher than in the low-trust context, as the high-trust context should be overwhelmingly populated by people willing to contribute to the public good^4,40^. In the low-trust context, on the other hand, contributions to the public good should be a rare occurrence, making free-riding a less promising strategy. Thus, the dilemma will be mostly overcome through individual solutions. As a consequence, we should observe that overall:

### H3a

*People who experienced a high-trust context will be more likely to reach the threshold for collective solutions than people who experienced a low-trust context.*

### H3b

*People who experienced a high-trust context will be more likely to have a higher coordination efficiency than people who experienced a low-trust context.*

### H3c

*People who experienced a low-trust context will be more likely to reach the threshold for individual solutions than people who experienced a high-trust context.*

As discussed by Nannestad, social trust is often conceptualized as either more relational and rational, or more moral, cultural, and dispositional^41^. According to the latter view, particularly associated with Uslaner, trust is “a general outlook on human nature” (p. 17)^42^. Given either conceptualization, the manipulation of social trust in the context of the iterated TG might not shape broader social trust. If trust is strictly relational (i.e., specific to certain trust objects) then individuals will not transpose anything learned about trustworthiness in the groups of playing iterated TG to the very different context of the broader social world. And, if trust is a deeper disposition, then it will not be amenable to change at all^43–45^.

Given that, we also test whether individuals will transpose their experiences during the iterated TG into beliefs about the wider real world, as found previously by Paxton and Glanville^46^ in support of the social learning perspective^7,41,47,48^. More precisely, we propose that experiencing a high- versus low-trust experimental context in an interactive experiment may—at least temporarily—influence people’s general optimism about human nature (consistent with one part of Uslaner’s theory) and potentially their mood, affect, and/or sense of control. Each of these things, according to a number of prior studies, are closely tied to trust, and amenable to experimental manipulation^49–51^. Insofar cooperation occurs because people tend to “assume that their own behavior is *diagnostic* of the behavior of others”^52^, then having just played a game in which they found themselves behaving in a more (less) trusting way, they should then possess more (less) optimistic expectations about the trustworthiness of generalized others in society.

We furthermore investigate whether assignment to high- versus low-trust contexts shapes participants’ views of measures for cooperative action in real-world environmental dilemmas—i.e., public policies for environmental protection. Many prior studies have found correlational evidence that individuals who report higher social trust are more supportive of environmental protection; do more privately to make their lifestyles environmentally benign; and comply more with environmentally consequential laws^12,53–55^. The literature suggests that generalized trust fosters environmental cooperation by raising individuals’ expectations about the benevolence and lawfulness of others^55^. When individuals expect others to obey rules and to contribute to environmental public goods, they are more likely to engage in such behaviors themselves^56^.

Such theoretical claims have not, however, been tested very rigorously in existing research. Most studies of the consequences of social trust for environmental attitudes and behaviors use observational data, with trust potentially confounded with one or more unmeasured other variables. We therefore take advantage of the exogenous manipulation in our experiment to test whether experiences of high- versus low-trust contexts shape participants’ responses to standard survey questions about social trust, and environmental attitudes that prior observational studies have found correlate with social trust.

We therefore hypothesize:

### H4

*People will give more trusting responses to standard survey questions about social trust.*

### H5a

*People who experience a high-social trust context will express more support for policies for environmental protection.*

### H5b

*Such people will report stronger intentions/plans to make a personal environmental effort.*

### H5c

*Such people will exhibit lower permissiveness towards environmental rule-breaking.*

Table 1 presents the list of all pre-registered confirmatory hypotheses and their expected relationships below.

**Table 1:** List of hypotheses and expected relationships.

	Hypothesis	Independent variable	Dependent variable	Hypothesized relationship
Behavioral outcomes	H1a	High-Trust (ref: Low-trust)	Invest in Collective solution	+
	H1b	Low-Trust (ref: High-trust)	Invest in Individual solution	+
	H2	High-Trust (ref: Low-trust)	Free-ride (invest in neither solution)	+
	H3a	High-Trust (ref: Low-trust)	Achieve threshold for Collective solution	+
	H3b	High-Trust (ref: Low-trust)	Coordination efficiency	+
	H3c	Low-Trust (ref: High-trust)	Achieve threshold for Individual solution	+
Attitudinal outcomes	H4	High-Trust (ref: Low-trust)	Social Trust	+
	H5a	High-Trust (ref: Low-trust)	Support for policies for env protection	+
	H5b	High-Trust (ref: Low-trust)	Willingness to make personal env effort	+
	H5c	High-Trust (ref: Low-trust)	Permissiveness towards env rule-breaking	−

## Results

**Fig. 1:**
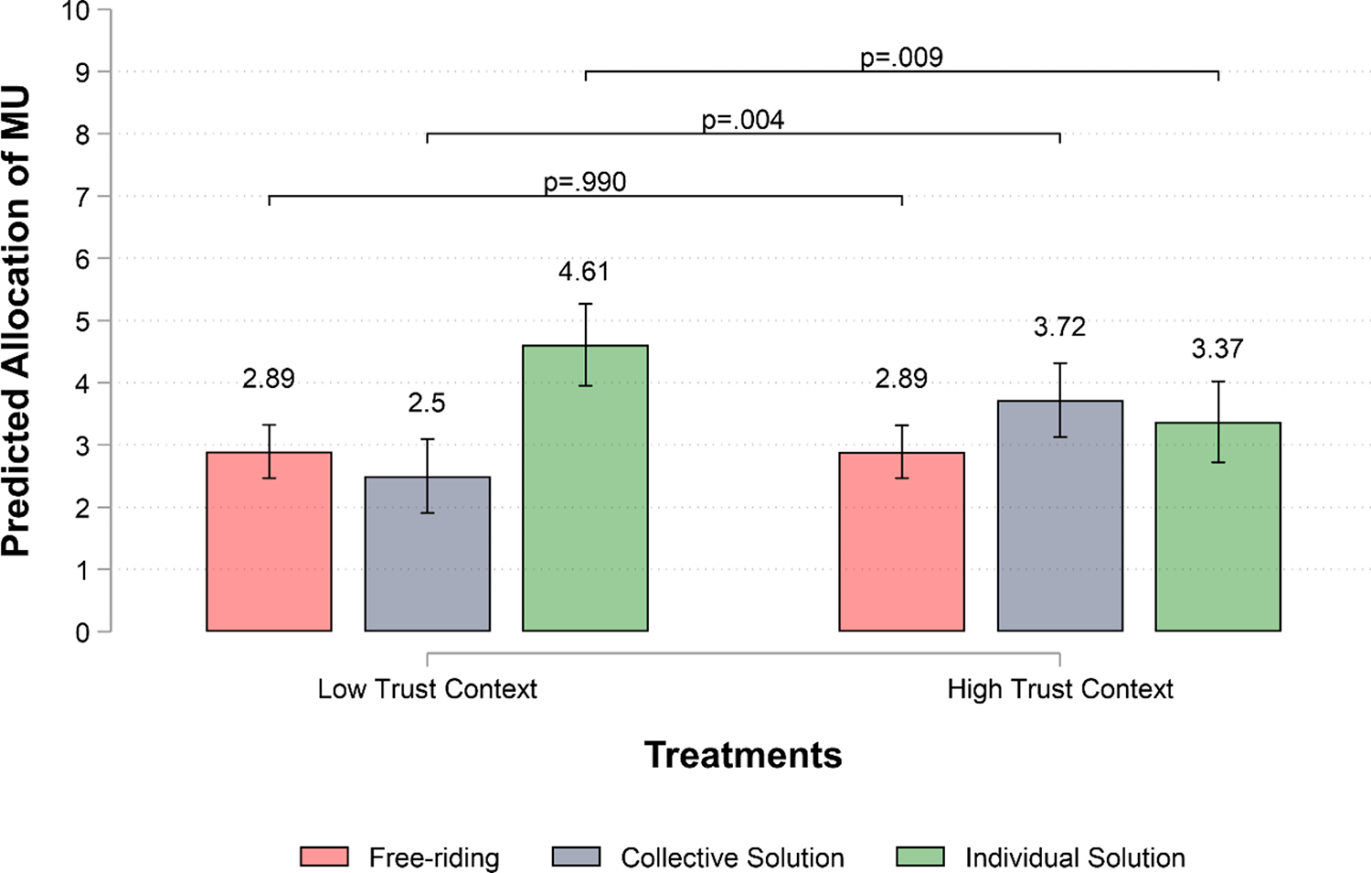
Multilevel linear models estimating the allocation of resources in each option by treatment. Predicted values for each decision by treatment with 95% CIs based on multilevel linear models with round-decisions nested in individuals nested in groups (Free-riding, N round-decisions/individuals/groups = 2,860/369/70; Collective Solution, N round-decisions/individuals/groups = 3,135/369/70; Individual Solution, N round-decisions/individuals/groups = 3,098/371/70). Results are robust to different statistical controls – see SM, tables B1.1–B1.3, B1.11–B1.14. We set statistical significance at the 5% level (i.e., α = 0.05) for two-sided tests. Notice that free-riding includes MUs individuals keep until either the collective or the individual solution is achieved. Investments in the collective solution include MUs allocated to the collective solution until this is achieved (group threshold = 240 MU). Investment in the individual solution include MUs allocated to the individual solution until this is achieved (individual threshold = 60 MUs).

Let us begin by presenting how participants responded to our manipulation. We find that subjects in the high-trust context are both more trusting and trustworthy: participants choose to trust 72% of the times in the high-trust context compared with 40% in the low-trust context (Trust_HT_ = 0.72, SE = 0.03; Trust_LT_ = 0.40, SE = 0.03; β_HT-LT_ = 0.32, SE = 0.05, *p* ≤ .001), and reciprocate trust 65% of the times compared with 46% in the low-trust context (Trustworthiness_HT_ = 0.65, SE = 0.04; Trustworthiness_LT_ = 0.46, SE = 0.04; β_HT-LT_ = 0.19, SE = 0.05, *p* ≤ .001) – see Supplementary Materials (SM), Figure A1. These results indicate that the manipulation works as expected. Moving to our main results, Fig. 1 illustrates the predicted values for each individual decision (i.e., MUs invested in the public solution, MUs invested in the private solution, and free-riding) by treatment (see also SM for an analysis of learning effects – Table C1 and Figure C1). On average, participants in the high-trust context invest more MUs in the collective solution (β = 1.221, SE = 0.428, *p* = .004) and less MUs in the individual solution (β = − 1.238, SE = 0.472, *p* = .009) than participants in the low-trust context, supporting H1a and H1b. On the other hand, we find no support for H2 since there is no statistically significant difference in free-riding behaviors between low- and high-trust contexts (β = − 0.004, SE = 0.308, *p* = .990). In other words, while high-trust contexts effectively stimulate investments towards collective solutions, they do not appear to be more vulnerable to free-riding than low-trust contexts, where, instead, individual solutions are preferred.

**Fig. 2:**
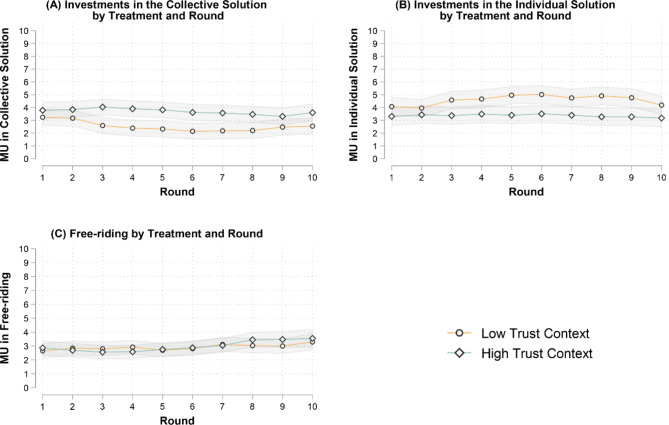
Multilevel linear models estimating the allocation of resources in each option by treatment and round. Panels (**A**)–(**C**) show the predicted values for each decision by round and treatment with 95% CIs based on multilevel linear models with round-decisions nested in individuals nested in groups (Free-riding, N round-decisions/individuals/ groups = 2,860/369/70; Collective Solution, N round-decisions/individuals/ groups = 3,135/369/70; Individual Solution, N round-decisions/individuals/ groups = 3,098/371/70). Results are robust to controls for age, education, gender, ethnicity, political orientation – see SM, tables B1.1–B1.3. We set statistical significance at the 5% level (i.e., α = 0.05) for two-sided tests. Notice that free-riding includes MUs individuals keep until either the collective or the individual solution is achieved. Investments in the collective solution include MUs allocated to the collective solution until this is achieved (group threshold = 240 MUs). Investment in the individual solution include MUs allocated to the individual solution until this is achieved (individual threshold = 60 MUs).

When focusing on individual decisions over rounds (see Fig. 2, Panels A–C), participants in the high- and low-trust contexts behave rather similarly at the beginning of the game. However, starting from the third round, participants in the two contexts begin to differ, with those in the low-trust context investing more heavily in the individual solution (Fig. 2, Panel B), lowering the investments in the public good (Fig. 2, Panel A) and therefore making the achievement of the collective threshold less and less likely. This suggests that, consistent with our theoretical argument, people in low-trust contexts lose faith in others’ willingness to contribute to the public good and reach the collective threshold. On the contrary, people in high-trust contexts continue relying on other participants, as their investments in the public good remain fairly stable throughout the game (Fig. 2, Panel A).

**Fig. 3:**
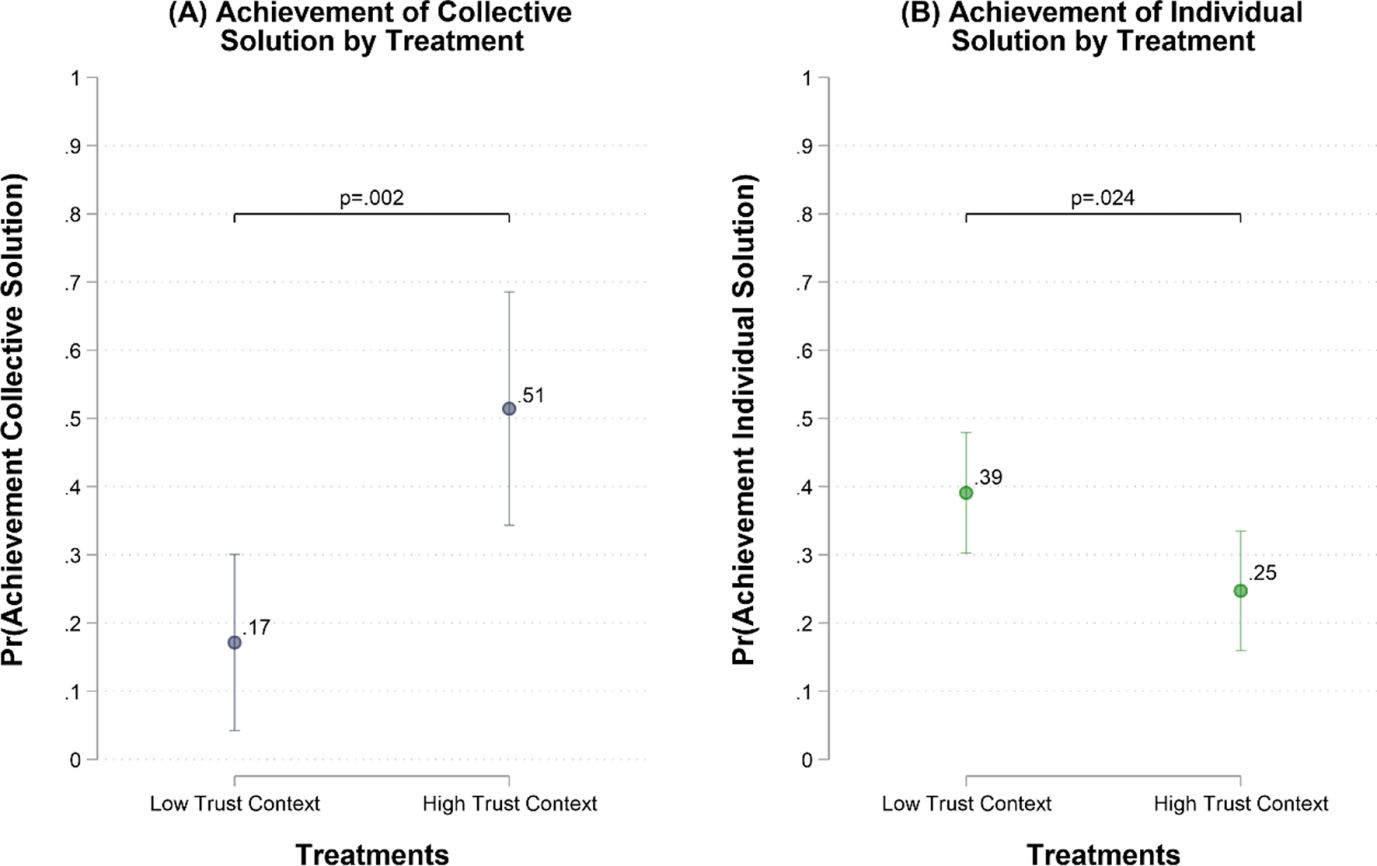
Linear models estimating the probability of achieving the collective and individual solution by treatment. Panel (**A**) shows the predicted probability of achieving the collective solution by treatment with 95% CIs based on a linear probability model with robust standard errors (R^2^ = 0.13). The unit of analysis is the group (*N* = 70). The collective solution is achieved when the group’s MUs investments in the collective solution reach the 240 MUs threshold. Panel (**B**) shows the predicted probability of achieving the individual solution by treatment with 95% CIs based on a multilevel linear probability model with individuals (*N* = 371) nested in groups (*N* = 70). The individual solution is achieved when the individual’s MUs investments in the individual solution reach the 60 MUs threshold. We set statistical significance at the 5% level (i.e., α = 0.05) for two-sided tests, and results in both panels are robust to different statistical controls and modelling strategies (e.g., multilevel logistic regression) – see SM, tables B1.4–B1.5, B1.9–B1.10.

Next, we evaluate whether observed differences in investments lead to a lower or higher probability to achieve collective and individual solutions across treatments. Figure 3, Panel A indicates that groups in the high-trust context are more likely to achieve the collective solution than groups in the low-trust context (β = 0.343, SE = 0.107, *p* = .002). Moreover, participants in the high-trust context are less likely to overcome the social dilemma by reaching the individual solution than participants in the low-trust context (β = − 0.144, SE = 0.064, *p* = .024) as shown in Fig. 3, Panel B. These findings provide support for H3a and H3c, showing that observed differences in investments lead to a higher chance to achieve collective solutions in the case of high-trust contexts (in comparison to low-trust contexts), and a higher chance to achieve individual solutions in low-trust contexts (in comparison to high-trust contexts).

**Fig. 4:**
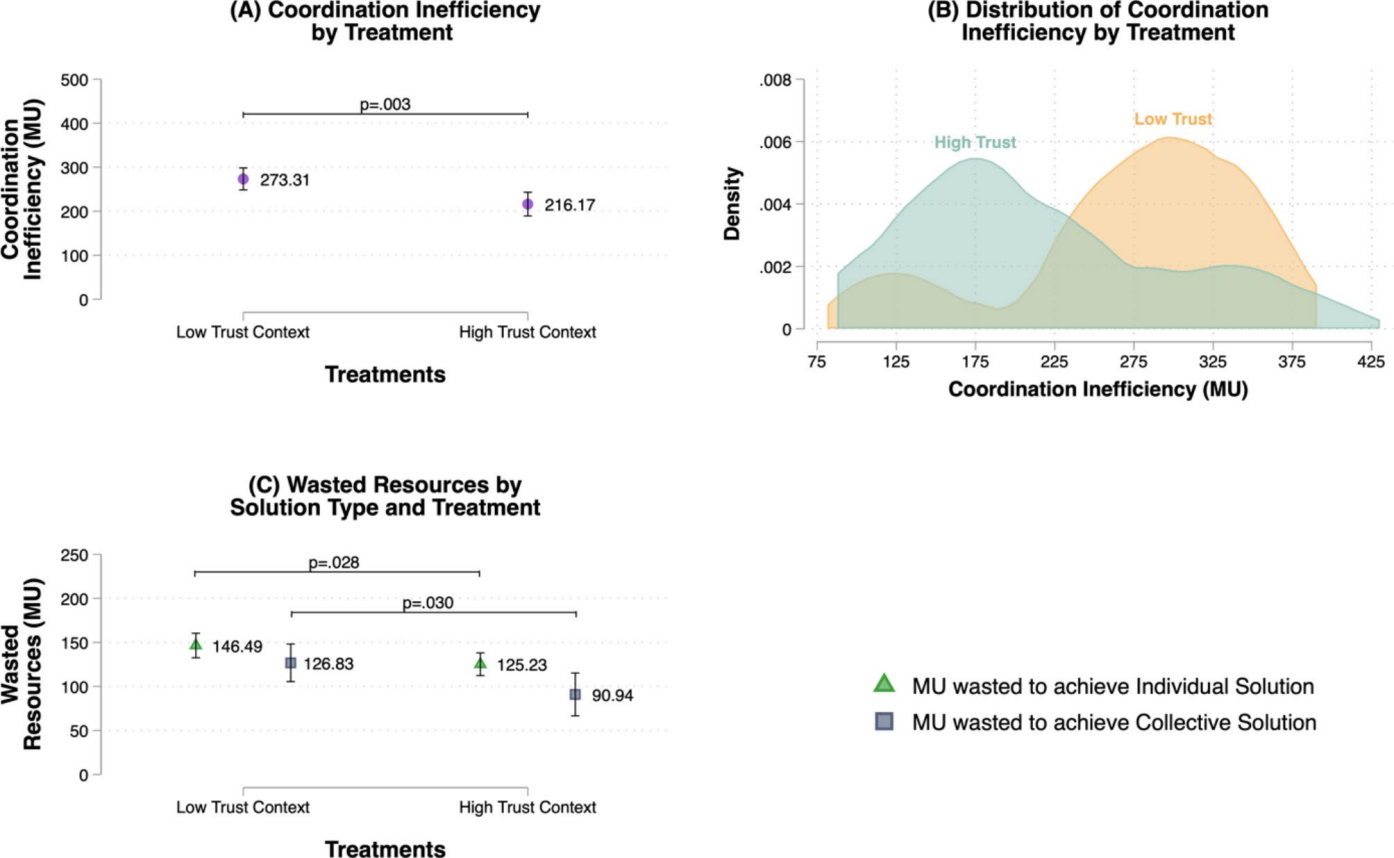
Linear models estimating the amount of coordination inefficiency and wasted resources by treatment and solution type. Panel (**A**) shows the predicted values of coordination inefficiency by treatment with 95% CIs based on an OLS regression model with robust standard errors (R^2^ = 0.12). The unit of analysis is the group (*N* = 70). Results are robust to different statistical controls – see SM, table B1.6. Coordination inefficiency is defined as MU investments that deviated from the optimal allocation of resources to achieve the collective solution. Panel (**B**) illustrates the distribution of coordination inefficiency in the high- and low-trust contexts. Panel C shows the predicted values of wasted resources to achieve the individual solution (R^2^ = 0.07) and the collective solution (R^2^ = 0.07) by treatment with 95% CIs based on OLS models with robust standard errors. The unit of analysis is the group (*N* = 70). Results are robust to different statistical controls – see SM, tables B1.7–B1.8. Wasted resources are defined as MU invested in the individual or collective solution that deviated from the optimal allocation of resources. We set statistical significance at the 5% level (i.e., α = 0.05) for two-sided tests.

Taken together, these findings indicate that both in low- and high-trust contexts people invest in the collective and the individual solutions. However, participants in low-trust contexts only occasionally achieve the collective solution and often opt to heavily invest in the individual solution as well, failing to coordinate on the most efficient option (i.e., the collective one – given the game’s parameters, achieving the individual solution costs 50% more than the collective solution, assuming that each participant contributes her fair share; see Materials and Methods). Consistent with H3b, this should lead us to expect that a larger amount of resources is wasted in low-trust contexts in comparison to high-trust contexts. Panels A and B in Fig. 4 illustrate, respectively, the predicted values and the distribution of coordination inefficiency (i.e., MUs investments that deviated from the optimal allocation of resources to achieve the collective solution) across treatments. In particular, Panel A shows that groups in high-trust contexts waste on average 57 MUs less than groups in the low-trust context (β = − 57.143, SE = 18.378, *p* = .003), supporting H3b. Panel C further illustrates the predicted values of wasted investments (i.e., MUs invested in the individual or collective solution that deviated from the optimal allocation of resources) by type of solution and treatment, indicating that groups in the high-trust contexts waste less resources in the attempt of reaching either type of solution (β_ind_ = − 21.257, SE = 9.490, *p* = .028; β_coll_ = − 35.886, SE = 16.182, *p* = .030). This suggests that groups in the low-trust environment are less able to use their resources efficiently when dealing with a collective action dilemma with multiple solutions (e.g., by allocating resources to the collective solution even though the likelihood of reaching the threshold becomes lower), producing poor investment choices and higher losses even in relation to the individual solution.

Overall, our findings suggest that high-trust contexts perform well in collective action dilemmas with multiple solutions and potential inefficiency. As people rely on others, they invest more substantially in the public good throughout the game, leading to higher chances of achieving the collective solution. Further, in our sample, high-trust contexts are not more vulnerable to free-riding than low-trust contexts, suggesting that people do not tend to opt for more exploitative strategies once a cooperative environment is established (in the first game, the iterated TG). On the other hand, people in low-trust contexts rely on other fellow participants only at the very beginning of the social dilemma and then overwhelmingly prefer to invest in the individual solution. This lack of trust in generalized others leads to a decrease in investments in the public good, while also producing a larger waste of resources and therefore lower returns. In other words, while collective action dilemmas with multiple solutions are managed in different ways in low- and high-trust contexts, the latter are more likely to achieve solutions that are more efficient for the community.

**Fig. 5:**
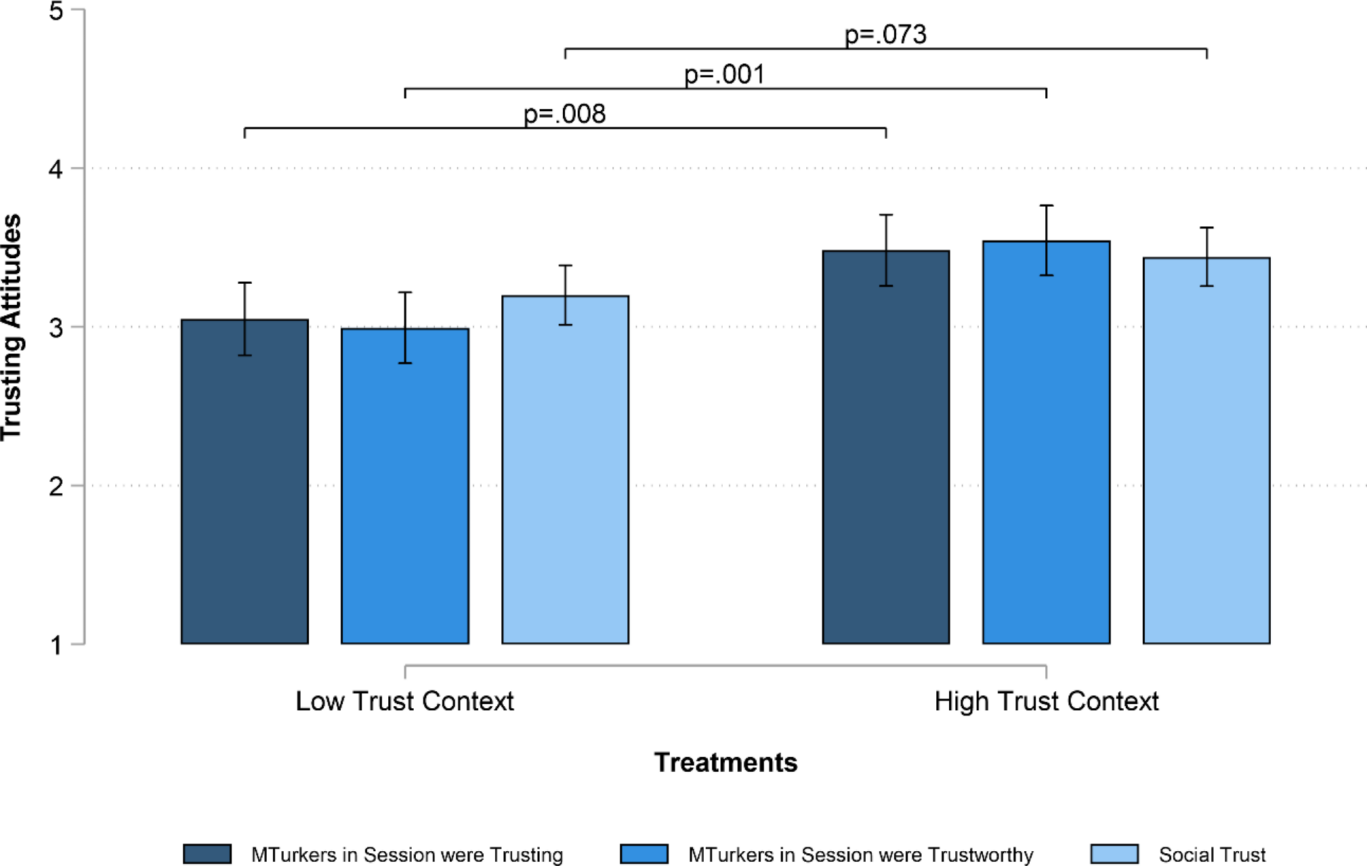
Multilevel linear models estimating trusting attitudes by treatment. Predicted values of trusting attitudes by treatment with 95% CIs based on multilevel linear models with individuals nested in groups (N individuals/groups = 371/70). Results are robust to different statistical controls – see SM, tables B2.1–B2.4. We set statistical significance at the 5% level (i.e., α = 0.05) for two-sided tests.

Finally, we turn to evaluate the impact of the treatment on our attitudinal outcomes. Figure 5 shows that people in the high-trust context report higher levels of perceived trust and trustworthiness of other anonymous MTurkers in their session (who are, as a matter of fact, unknown individuals) than people in the low-trust context (β_MTurkTrst_ = 0.434, SE = 0.163, *p* = .008; β_MTurkTrstwrth_ = 0.550, SE = 0.160, *p* = .001). However, the treatment affects responses to the standard survey measure of social trust only at the 0.10 level (β_SocTrust_ = 0.240, SE = 0.134, *p* = .073), providing no support for H4. Similarly, when we examine the impact of the treatment on support for policies for environmental protection (β_LessPoll_ = 0.209, SE = 0.107, *p* > .05; β_IncreaseTax_ = − 0.061, SE = 0.140, *p* > .05), willingness to make personal environmental efforts (β_UselessEnergy_ = 0.115, SE = 0.105, *p* > .05; β_RecycleMore_ = 0.093, SE = 0.103, *p* > .05; β_SaveWater_ = 0.026, SE = 0.117, *p* > .05), and permissiveness towards environmental rule-breaking (β_AcceptLittering_ = 0.151, SE = 0.204, *p* > .05; β_AcceptNoRecycle_ = − 0.088, SE = 0.152, *p* > .05), we find no statistically significant effects – providing no support for H5 (see SM, Section B2 for more details). This suggests that our manipulation did not change participants’ wider attitudes, and experiences within the context of the game did not spill over or transpose to general beliefs about the real world.

## Discussion

Our experimental study contributes to the literature on the behaviors of individuals confronted with collective action dilemmas. We investigate an issue that has previously received limited attention: the impact of (dis)trusting and (un)trustworthy contexts on public goods provision when participants can opt for both collective and individual solution to a shared problem. As some major social environmental challenges (e.g., CO_2_ reduction) can be understood as such dilemmas, the question of how people behave in these situations is an important one.

We find that in high-trust contexts participants tend to contribute to collective solutions, while in low-trust contexts they opt for individual solutions, thereby undermining the realization of collectively preferable outcomes. These results also have implications for coordination efficiency and resource wastage. In comparing the performance of high- and low-trust groups, we find significant inefficiencies in low-trust contexts. On the other hand, free-riding is equally common in the high- and low-trust groups. High-trust communities therefore appear well-equipped to deal with complex dilemmas, and low-trust communities manage to overcome such dilemmas, albeit while wasting more resources, without suffering complete coordination failure. From a policy perspective, our findings suggest that it may be appropriate to design policies that (a) facilitate the development of trust (e.g., by increasing the potential benefits of mutual cooperation among unknown individuals) to help coordination in overcoming collective action dilemmas and/or (b) support different types of solutions depending on the pre-existing trust levels of the targeted community. For instance, developing on the latter point, to lower CO_2_ emissions produced by commuting in a low-trust community, policymakers could consider incentivizing more substantially private solutions (e.g., purchasing electric vehicles) rather than a collective one (e.g., public transport use). Indeed, even if the overall efficiency of private solutions is lower than collective ones, having stronger incentives to opt for the private solution should lower the misuse of resources and allow the community to address, at least to some extent, the shared problem – e.g., without wasting resources on an unsatisfactory or inadequate outcome on the collective solution. On the other hand, in high-trust communities incentivizing a collective solution (e.g., public transport use) rather than private solutions (e.g., purchasing electric vehicles) may be a more promising strategy given the ability of high-trust communities to efficiently solve coordination problems.

Our study also explores the role of trust in cooperative decision-making in other ways. Our manipulation does not produce broad shifts in self-reports of participants’ social trust, though in high-trust contexts we do find that participants reported higher trust in strangers involved in the study. This finding seems to suggest that responses to standard social trust measures are not subject to transient influences. In addition, the varying trust contexts experienced in our study did not make any discernible difference to participants’ support for real-world environmental policies, suggesting that the manipulation did not extend to broader attitudes and policy preferences. It may be that social trust does not in fact influence environmental policy support, contrary to some prior studies^57^. Potentially, environmental policy attitudes are instead a function of people’s political or institutional trust. Alternatively, the specific manipulation applied in this study may simply not have been strong enough to have much impact on social trust. Or, social trust may be resilient not only to manipulation in a lab setting, but even more broadly, in the social world. Bauer for example has previously found little evidence that trust responds even to notable life events^58^, and Fairbrother et al. found that fluctuations in social trust due to experiences of corruption made only a short-lived impact on people’s trust^59^. Future research would do well to investigate these possibilities further by, for example, creating trusting environments with alternative approaches (e.g., manipulating the composition of groups in relation to pre-existing trusting attitudes), aiming at disentangling how contextual features produce the observed changes in behaviors and identify which attitudes are most affected.

## Materials and methods

### Participants and general setup

We ran the experiment between 11th February 2022 and 31st March 2022 using oTree (version 3.4.0), which offers an integration with Amazon Mechanical Turk (AMT)^60^. This let us conduct the interactive experiment through oTree’s interface, recruiting participants from AMT. To help ensure high quality data, we recruited MTurkers who have a certified and long-lasting history of consistent work, restricting our sample to U.S. participants^61–64^.

Consistent with current standards in the literature and following previous studies in the field assessing the same decision-making tasks^22–24^, we gathered data for at least 30 groups per treatment (with each group having a planned number of 6 participants) reaching a sample size of 371 participants across 70 groups (please see SM, Tables A1–A2 for descriptive statistics and sample characteristics).

Before running the study, ethical approval was granted by the University College London, Institute of Education Ethics Review Committee (REC1575). Written informed consent was obtained from all participants at the beginning of each experimental session. Participants were informed that they were taking part in a research study and completed the consent form. There was no deception of respondents. All research was performed in accordance with relevant guidelines and regulations. Hypotheses and data analysis plan were pre-registered on the Open Science Framework (see https://osf.io/qud6k/?view_only=de060718e56b4eccac095359c0ace1c0) before data collection.

## Experimental design

**Fig. 6:**
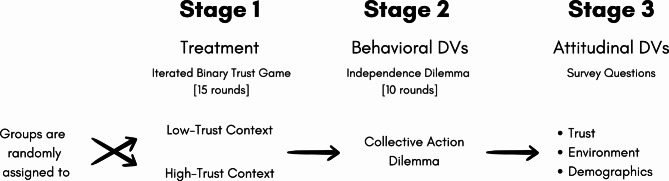
Overview of the experimental design.

As Fig. 6 illustrates, the study consists of three stages: (Stage 1) instructions and 15 rounds of the binary TG (in addition to 2 trial rounds to allow participants to familiarize themselves with the game), (Stage 2) instructions and 10 rounds of the Independence Dilemma, and (Stage 3) a follow-up survey to gather background and attitudinal information^4,22–24^.

Participants first play the iterated TG for 15 rounds. In the TG, there are two roles: the Truster and the Trustee. Within each round, the Truster is given an endowment in experimental points and has the choice to send or keep the endowment. If the Truster sends her endowment, the researcher multiplies the sum sent by a fixed amount. Then, the Trustee can decide whether to keep the sum received or return part of it to the Truster. The action of the Truster implies a trusting behavior, while the action of the Trustee implies a trustworthy behavior – see Banerjee, et al.^65^ for recent empirical evidence using representative samples across the UK, USA, France, Italy, Germany, Korea, and Slovenia showing a robust correlation between attitudinal measures of social trust and behavioral measures of the same concept. Roles are randomly assigned at the beginning of the session and are fixed throughout the game.

As mentioned above, we aim to investigate which factors favor collective solutions over individual ones or free-riding, and in which conditions inefficient allocation of resources is minimized. We particularly focus on (dis)trusting and (un)trustworthy contexts in identifying the factors underpinning collective action. Thus, at the beginning of stage 1 each group is randomly assigned to one of the following treatments: (1) *low-trust context* where the payoffs in the binary TG are set to reduce the likelihood of trusting and trustworthy behaviors or (2) *high-trust context*, in which the payoffs in the binary TG are set to increase the likelihood of trusting and trustworthy behaviors. Participants are not informed of the existence of the other treatment.

**Fig. 7:**
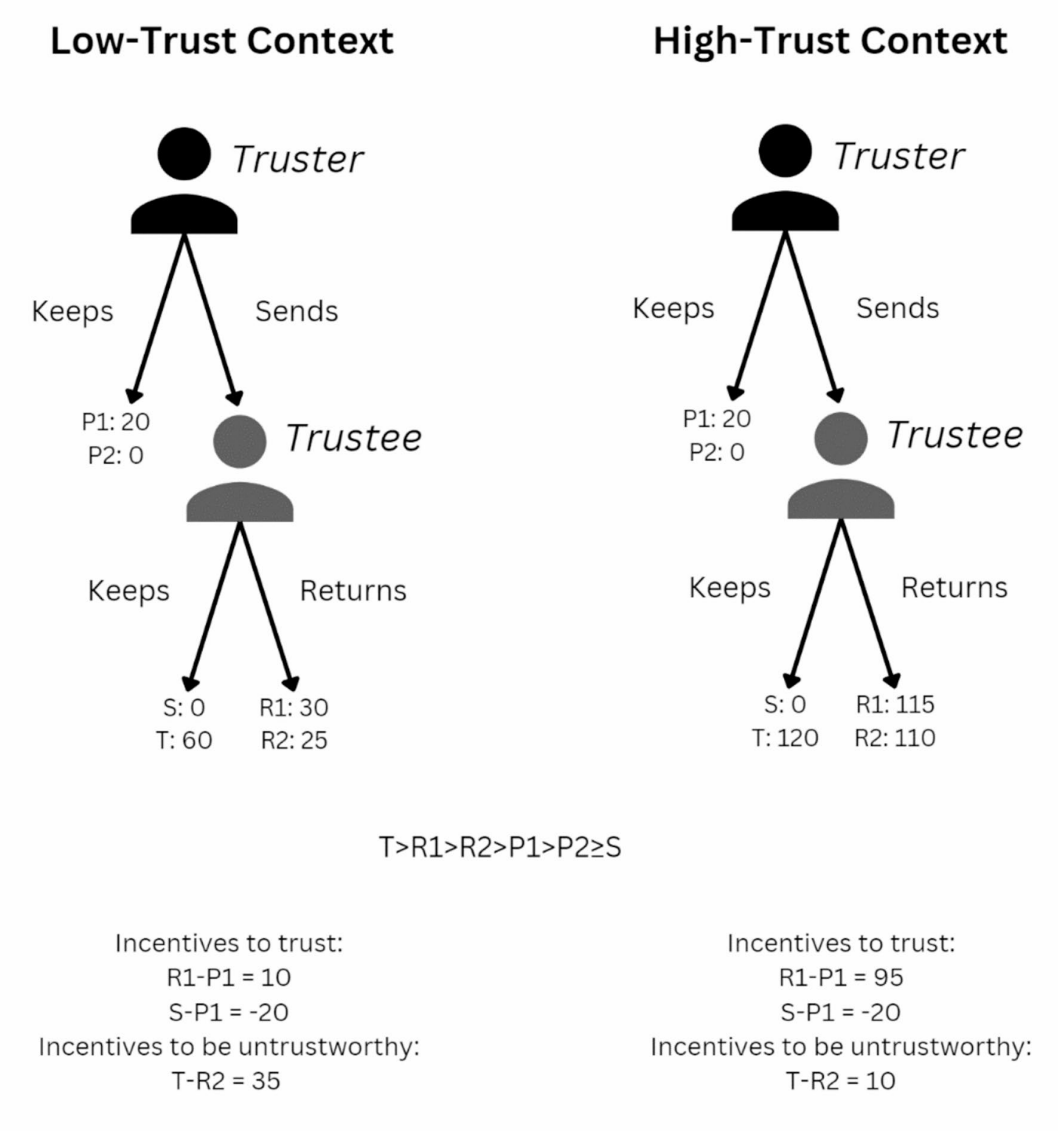
Treatments and payoffs in the binary trust game.

The incentives to trust are 9.5 times higher in the high-trust than the low-trust context, while the incentives to be untrustworthy are 3.5 times lower in the high-trust than the low-trust context^4^. In addition, the low-trust context has lower incentives to cooperate than the standard version of the game^26^. Nevertheless, both treatments maintain the essential premises of a trust situation since they respect the condition T > R_1_ > R_2_ > P_1_ > P_2_ ≥ S (see Fig. 7)^66,67^.

Thus, trusting and trustworthy behaviors are encouraged in dyadic interactions in the high-trust context and discouraged in the low-trust context. Furthermore, to give subjects a perception of the general trend in the group, we let participants know the percentage of players who trusted or reciprocated in the group at the end of each round. However, no information on individual history of players is provided. Participants are matched with a different partner each round and are not informed of the exact number of players involved in the session. After the iterated binary TG, the experimental points earned are converted into dollars and participants are shown their total earnings. To avoid strong variations in earnings across treatments, we apply different conversion rates for the points earned in the TG in the low-trust and high-trust context so that the distribution of earnings after the TG would be similar across treatments (between $4 and $6). Participants are not informed of the conversion rate of experimental points into dollars before the start of the experiment in either treatment to avoid triggering dissimilar reactions^4^.

**Fig. 8:**
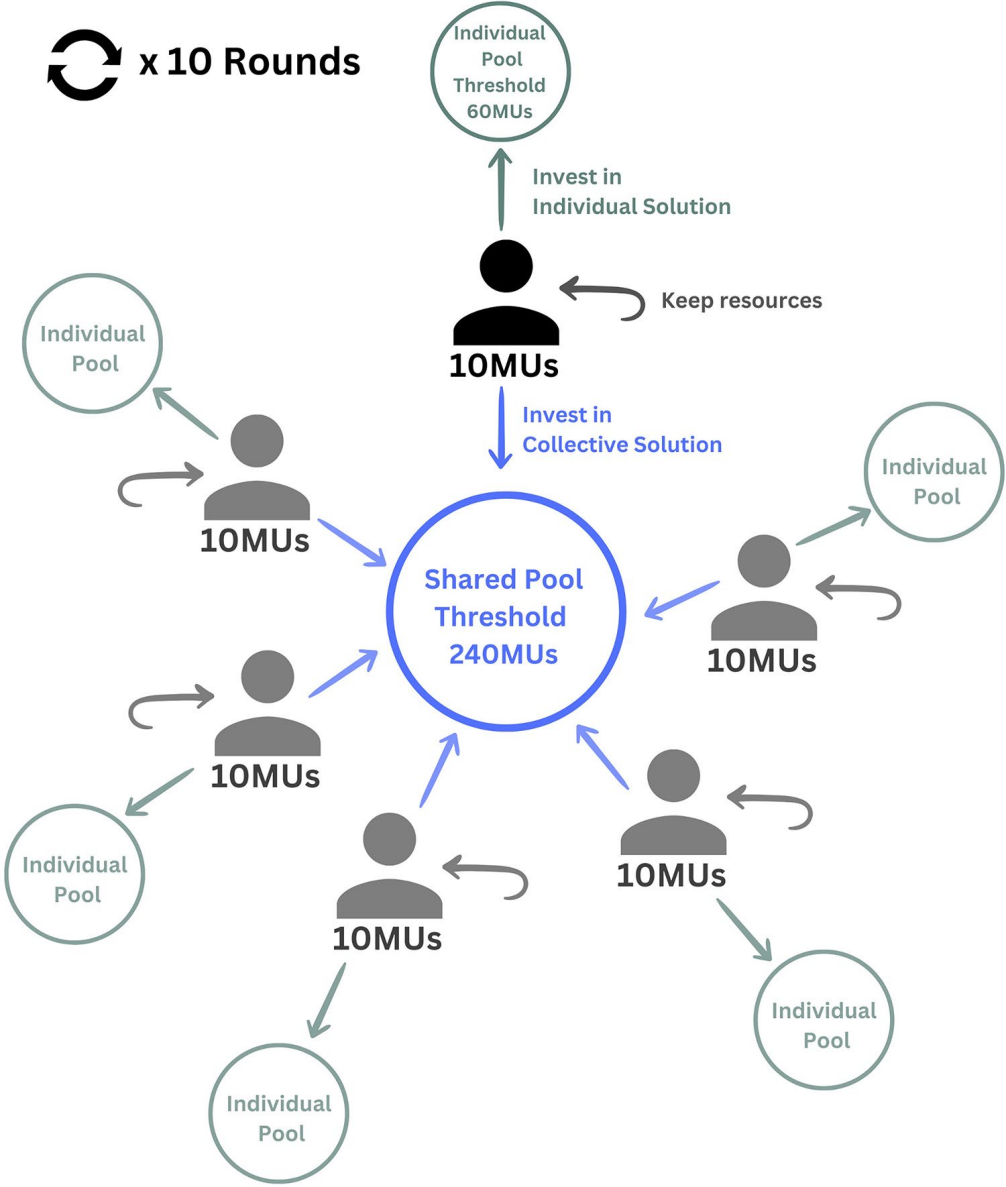
Participants’ round choices in the Independence Dilemma.

In stage 2, participants play the Independence Dilemma for 10 rounds^23^ (see Fig. 8). In the Independence game, participants play as part of a group. In every round, each group member receives 10 Monetary Units (MUs) and needs to decide how to allocate them. Participants are informed of the conversion rate of MUs into dollars (10 MUs = $1) at the beginning of the stage. Each group member can allocate the MUs in a private individual pool, in a shared public pool, or keep any amount of MUs for herself. Private pools are separated for each member, while the public pool is a shared account where all group members can contribute. After the final round, each participant can keep any MU not invested (i.e., MUs kept for themselves during the game) if (1) the group allocated enough MUs to the public pool and reached a predefined threshold (240 MUs) or (2) the participant allocated enough MUs to her private pool and reached a predefined threshold (60 MUs). If participants did not reach either the private or public threshold by the last round, they lose all MUs. If the threshold for the shared public pool is reached, all participants can keep the MUs not invested regardless of their contribution to the shared public pool. On the other hand, reaching the threshold for the private pool allows only the participant who achieved that threshold to keep the MUs not invested during the game (not the entire group). After each round, each group member is informed of the amount of MUs accumulated in their private and public pool as well as the allocation decisions of the other group members.

In other words, the Independence game is a collective action dilemma, in which each participant has three options: invest in individual solutions; invest in collective solutions; or free-ride (invest in neither solution). If participants do not invest enough to achieve either solution, then any resources they have held back are lost. Their investment strategy will therefore reflect their expectations about what other participants will do. The co-existence of individual and collective solutions allows us to study social dilemmas particularly vulnerable not only to free-riding but also to coordination failure and inefficiency.

Gross and De Dreu show that the decision to opt for individual or collective solutions depends on their relative cost^23^. If individual solutions are more costly to the individual in comparison to collective ones, then collective solutions are more likely to be chosen. That is, if the cost-benefit ratio of individualism is high, as it seems to be in social dilemmas concerning the environment, then opting for a collective solution would be more advantageous^68,69^. Thus, to reproduce a collective action dilemma concerning an environmental issue, we implement an Independence Dilemma with a cost-benefit ratio of individualism equal to 1.5, meaning that achieving the individual solution is 50% more expensive than achieving the collective solution assuming that each participant contributes her fair share (see Gross and De Dreu for further discussions^23^).

In stage 3, participants respond to a questionnaire covering a variety of topics concerning social, particular, and political trust, as well as environmental attitudes, risk preferences, and socio-demographics.

## Measures

We have 6 behavioral and 4 attitudinal outcome variables of interest for the pre-registered confirmatory analysis. The behavioral outcomes are the following: (B1) the investment in the individual solution at each round (0–10 MUs); (B2) the investment in the collective solution at each round (0–10 MUs); (B3) free-riding at each round (0–10 MUs); (B4) the achievement of the individual solution threshold (1 = individual solution threshold achieved, 0 = otherwise); (B5) the achievement of the collective solution threshold (1 = collective solution threshold achieved, 0 = otherwise); and (B6) a continuous variable measuring coordination inefficiency. Free-riding includes MUs individuals keep until either the collective or the individual solution is achieved. Investments in the collective solution include MUs allocated to the collective solution until this is achieved. Investment in the individual solution include MUs allocated to the individual solution until this is achieved. Coordination inefficiency is defined as MU investments that deviated from the optimal allocation of resources to achieve the collective solution^23^.

The attitudinal outcome variables are: (A1) social trust (1 = you can’t be too careful, 5 = most people can be trusted); (A2) support for policies for environmental protection (1 = no support at all, 5 = complete support); (A3) willingness to make personal environmental effort (1 = not likely at all to make changes, 5 = very likely to make changes); (A4) permissiveness towards environmental rule-breaking (1 = never acceptable, 5 = always acceptable).

### Analytical strategy

To estimate the average treatment effect on our outcome variables at the round-decision level B1–B3, we employ multilevel linear regression models accounting for the lack of independence among observations with round-decisions nested in individuals nested in groups. For outcome variables at the individual level B4, A1-A4, we run multilevel linear regression models with individuals nested in groups. Finally, for outcome variables at the group level (B5–B6), we use linear probability models. We set statistical significance at the 5% level (i.e., α = 0.05) for two-sided tests. As part of our exploratory analysis and robustness checks, we adjust for round effects and baseline covariates potentially unevenly distributed among groups (e.g., age, gender, education, ethnicity, and political conservatism), as well as estimating the evolution of participants’ behaviors over rounds by treatment (i.e., looking at the interaction between treatment and round). Last but not least, we further check the robustness of our findings by adopting different modeling strategies (e.g., logistic regression model and multilevel logistic regression model – see SM, Section B).

## Electronic supplementary material

Below is the link to the electronic supplementary material.

Supplementary Material 1

## Data Availability

All data and materials generated or analyzed during this study are available on the Open Science Framework (OSF) repository (https://osf.io/qud6k/?view_only=de060718e56b4eccac095359c0ace1c0).
