# Supplementary material for: The effect of trusting contexts in social dilemmas with collective and individual solutions
Source: Sci Rep. 2024 Oct 30;14:26048. doi: 10.1038/s41598-024-77190-3 (PMC11522509; doi:10.1038/s41598-024-77190-3)
Supplement: Supplementary file 1 — Supplementary Material 1 [file 41598_2024_77190_MOESM1_ESM.docx]

**Supplementary Information**

The Effect of Trusting Contexts in Social Dilemmas with Collective and Individual Solutions

*Section A – Descriptive statistics and manipulation effectiveness2*

*Section B – Regression models and robustness checks5*

*B1 – Behavioral outcomes (H1-H3) 5*

*B2 – Attitudinal outcomes (H4-H5) 16*

*Section C – Learning effects18*

**Section A – Descriptive statistics and manipulation effectiveness**

## *Participants recruitment and qualification criteria*

The experiment was conducted between 11^th^ February 2022 and 31^st^ March 2022. The experiment was programmed in oTree (version 3.4.0) (Chen, Schonger and Wickens 2016), which allowed us to advertise the experiment sessions on Amazon Mechanical Turk (AMT) from the oTree’s interface and pay participants directly through the AMT system. We recruited MTurkers who have a certified and long-lasting history of consistent work (i.e., 95% or more of previous assignments approved and participation in at least 1000 past assignments), restricting our sample to U.S. participants (Cheung et al. 2017; Moss et al. 2020; Peer, Vosgerau and Acquisti 2014; Robinson et al. 2019). We published the experimental sessions between 11:00 and 17:00 EST. In addition to standard pre-screening strategies, we registered MTurkers’ ID after each session to prevent retakes.

Participants were paid a fixed reward of $1.00 as a showing up fee. To limit dropouts during the game, we rewarded participants for the time they spent in the waiting room, where they stayed for a maximum of 10 minutes until the planned number of participants for each session (6 people) was reached. Participants were paid $0.01 for every five seconds of waiting time in the waiting room. To make sure that all participants earned a minimum of $5.5 by the end of the study, participants who earned less than $5.5 in total at the end of games were additionally compensated in the post-experiment survey by topping up their earnings to $5.5. Participants earned on average $6.43 (SD=1.24). Given that the experiment lasted about 40 minutes on average, the minimum payment of $5.5 guaranteed that all participants were paid at an hourly pay rate higher than the US federal minimum wage of $7.25.

When participants did not make a decision in the Trust Game, we allowed the computer to act on their behalf to avoid frozen sessions. The computer was set to defect (i.e., not trust or not reciprocate) in the Low Trust context, and to cooperate (i.e., trust or reciprocate) in the High Trust context, therefore avoiding bias within treatments. Nonetheless, in the Low Trust context we let the computer to occasionally trust to allow human trustees to play more frequently. When participants did not make a decision in the Independence Dilemma, we again allowed the computer to act on their behalf to avoid frozen sessions. The computer was set to act randomly in this instance to avoid bias. Non-human actions were recoded as missing.

Experimental sessions were considered failed if (a) more than three participants in the session were idle in the first three rounds of the Independence Dilemma and did not read the instructions or failed the comprehension questions; (b) human actions in the session were less than 60% across all rounds of the Independence Dilemma.

In a similar vein, participants who did not play or read the instructions for the Trust Game or the Independence Dilemma were considered unreliable and discarded for the analysis. Including or excluding such observations does not significantly change the results of the study (see tables B1.11-B1.13).

**Table A1.** Sample characteristics

| Variables | mean | sd | min | max | N ind. |
| --- | --- | --- | --- | --- | --- |
| Gender | 0.41 | 0.49 | 0 | 1 | 371 |
| Age | 37.71 | 10.75 | 20 | 74 | 371 |
| Education | 0.78 | 0.42 | 0 | 1 | 371 |
| Ethnicity | 0.86 | 0.35 | 0 | 1 | 371 |
| Conservative Scale | 2.99 | 1.31 | 1 | 5 | 371 |

**Note.** N ind. = N individuals. Gender (1=Female, 0=Male); Education (1=Associate degree or more; 0=Less than associate degree); Ethnicity (1=White, 0=Other); Conservative Scale (1=Very Liberal, 5=Very Conservative).

**Table A2.** Descriptive statistics of MU allocation by treatment

|  | mean | sd | min | max | N ind. |
| --- | --- | --- | --- | --- | --- |
| *Low Trust Context* |  |  |  |  |  |
| Public Solution | 2.53 | 2.23 | 0 | 10 | 181 |
| Individual Solution | 4.62 | 3.20 | 0 | 10 | 182 |
| Free-riding | 2.87 | 2.47 | 0 | 10 | 181 |
| *High Trust Context* |  |  |  |  |  |
| Public Solution | 3.72 | 3.02 | 0 | 10 | 188 |
| Individual Solution | 3.41 | 3.33 | 0 | 10 | 189 |
| Free-riding | 2.87 | 2.71 | 0 | 10 | 188 |

**Note.** N ind. = N individuals.

## *Manipulation effectiveness*

Figure A1 shows the effectiveness of our manipulation in the two treatments, clearly indicating that participants in the high trust context were significantly more likely to act in a trusting (β=.32, SE=.05, p≤0.001) and trustworthy (β=.18, SE=.05, p≤0.001) manner than participants in the low trust context.

**Figure A1.** Manipulation effectiveness by treatment


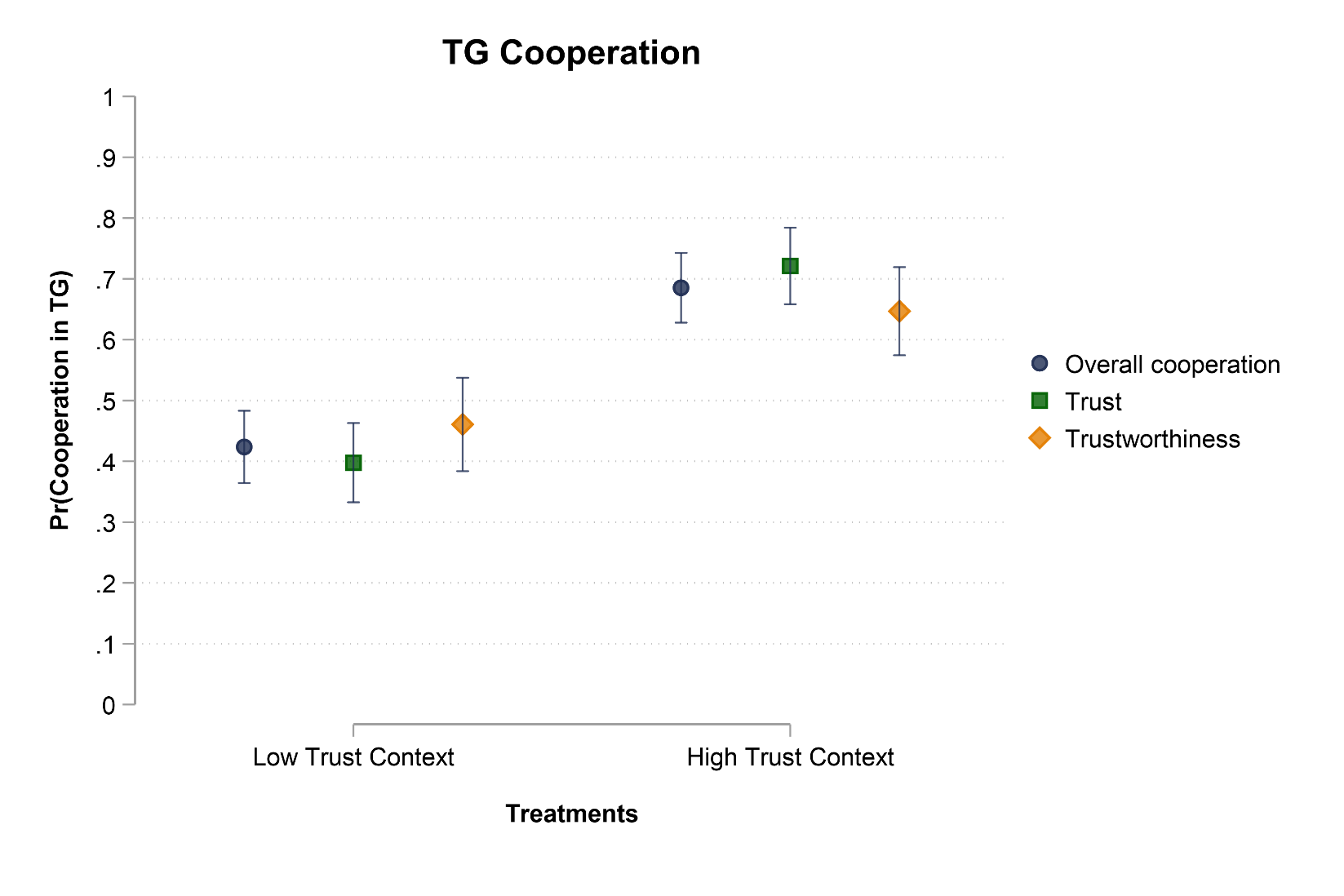


**Note.** Predicted values for each decision by treatment with 95% CIs based on multilevel linear models with round-decisions nested in individuals nested in groups (Trust, N round-decisions/individuals/ groups = 2,991/185/70; Trustworthiness, N round-decisions/individuals/ groups = 1,692/183/69).

**Section B – Regression models and robustness checks**

Here we present results from regression models estimating the impact of the treatment on the behavioral and attitudinal outcomes of interest discussed in the main text. Further, we check the robustness of our findings by adjusting for round effects and potentially uneven distribution of relevant baseline covariates across treatments, considering different modeling strategies (e.g., logistic regressions and multilevel logistic regressions), and including participants who did not play or read the instructions for the Trust Game or the Independence Dilemma. As illustrated below, results from the robustness checks are in line with results shown in the main text.

*B1. Behavioral outcomes (H1-H3)*

**Table B1.1.** Multilevel linear models estimating public solution investments without and with controls.

| DV: | Public Solution  Model 1 | | Public Solution  Model 2 | | Public Solution  Model 3 | |
| --- | --- | --- | --- | --- | --- | --- |
| *Treatment (ref: Low Trust)* |  |  |  |  |  |  |
| High Trust | 1.221** | (0.428) | 1.246** | (0.423) | 0.589 | (0.491) |
| Age |  |  | 0.004 | (0.012) | 0.004 | (0.012) |
| *Education (ref: Less than Associate degree)* |  |  |  |  |  |  |
| Associate degree or more |  |  | -0.175 | (0.298) | -0.178 | (0.298) |
| *Gender (ref: Male)* |  |  |  |  |  |  |
| Female |  |  | -0.051 | (0.249) | -0.050 | (0.249) |
| *Round (ref: round 1)* |  |  |  |  |  |  |
| Round 2 |  |  | -0.001 | (0.173) | -0.064 | (0.246) |
| Round 3 |  |  | -0.186 | (0.172) | -0.644** | (0.244) |
| Round 4 |  |  | -0.356* | (0.171) | -0.850*** | (0.243) |
| Round 5 |  |  | -0.431* | (0.171) | -0.914*** | (0.242) |
| Round 6 |  |  | -0.626*** | (0.172) | -1.092*** | (0.242) |
| Round 7 |  |  | -0.634*** | (0.174) | -1.057*** | (0.244) |
| Round 8 |  |  | -0.673*** | (0.175) | -1.033*** | (0.245) |
| Round 9 |  |  | -0.586** | (0.179) | -0.763** | (0.244) |
| Round 10 |  |  | -0.415* | (0.186) | -0.692** | (0.250) |
| High Trust × Round 2 |  |  |  |  | 0.120 | (0.345) |
| High Trust × Round 3 |  |  |  |  | 0.902** | (0.342) |
| High Trust × Round 4 |  |  |  |  | 0.971** | (0.341) |
| High Trust × Round 5 |  |  |  |  | 0.952** | (0.340) |
| High Trust × Round 6 |  |  |  |  | 0.929** | (0.343) |
| High Trust × Round 7 |  |  |  |  | 0.845* | (0.347) |
| High Trust × Round 8 |  |  |  |  | 0.717* | (0.350) |
| High Trust × Round 9 |  |  |  |  | 0.288 | (0.358) |
| High Trust × Round 10 |  |  |  |  | 0.511 | (0.376) |
| *Ethnicity (ref: Other)* |  |  |  |  |  |  |
| White |  |  | -0.361 | (0.358) | -0.363 | (0.359) |
| Conservative Scale |  |  | 0.152 | (0.095) | 0.152 | (0.095) |
| Constant | 2.500*** | (0.303) | 2.756*** | (0.672) | 3.091*** | (0.685) |
| Round-decisions/Individuals/Sessions | 3,135/369/70 | | 3,135/369/70 | | 3,135/369/70 | |
| SD individuals | 2.033 | (0.094) | 2.030 | (0.094) | 2.035 | (0.095) |
| SD session | 1.520 | (0.179) | 1.467 | (0.179) | 1.484 | (0.180) |

**Note.** Standard errors in parentheses. Significance levels: * p<0.05, **p<0.01. ***p<0.001

**Table B1.2.** Multilevel linear models estimating individual solution investments without and with controls.

| DV: | Individual Solution  Model 1 | | Individual Solution  Model 2 | | Individual Solution  Model 3 | |
| --- | --- | --- | --- | --- | --- | --- |
| *Treatment (ref: Low Trust)* |  |  |  |  |  |  |
| High Trust | -1.238** | (0.472) | -1.242** | (0.472) | -0.775 | (0.535) |
| Age |  |  | -0.008 | (0.015) | -0.008 | (0.015) |
| *Education (ref: Less than Associate degree)* |  |  |  |  |  |  |
| Associate degree or more |  |  | -0.345 | (0.386) | -0.344 | (0.386) |
| *Gender (ref: Male)* |  |  | 0.000 |  |  |  |
| Female |  |  | 0.140 | (0.321) | 0.144 | (0.321) |
| *Round (ref: round 1)* |  |  |  |  |  |  |
| Round 2 |  |  | 0.009 | (0.175) | -0.113 | (0.248) |
| Round 3 |  |  | 0.285 | (0.173) | 0.511* | (0.246) |
| Round 4 |  |  | 0.388* | (0.173) | 0.594* | (0.246) |
| Round 5 |  |  | 0.488** | (0.172) | 0.891*** | (0.245) |
| Round 6 |  |  | 0.570*** | (0.172) | 0.948*** | (0.245) |
| Round 7 |  |  | 0.386* | (0.178) | 0.684** | (0.253) |
| Round 8 |  |  | 0.392* | (0.181) | 0.842** | (0.259) |
| Round 9 |  |  | 0.322 | (0.185) | 0.699** | (0.266) |
| Round 10 |  |  | 0.006 | (0.189) | 0.125 | (0.277) |
| High Trust × Round 2 |  |  |  |  | 0.244 | (0.349) |
| High Trust × Round 3 |  |  |  |  | -0.445 | (0.345) |
| High Trust × Round 4 |  |  |  |  | -0.407 | (0.345) |
| High Trust × Round 5 |  |  |  |  | -0.793* | (0.344) |
| High Trust × Round 6 |  |  |  |  | -0.744* | (0.343) |
| High Trust × Round 7 |  |  |  |  | -0.586 | (0.355) |
| High Trust × Round 8 |  |  |  |  | -0.869* | (0.361) |
| High Trust × Round 9 |  |  |  |  | -0.727* | (0.369) |
| High Trust × Round 10 |  |  |  |  | -0.251 | (0.378) |
| *Ethnicity (ref: Other)* |  |  |  |  |  |  |
| White |  |  | 0.227 | (0.462) | 0.236 | (0.463) |
| Conservative Scale |  |  | -0.273* | (0.122) | -0.275* | (0.122) |
| Constant | 4.609*** | (0.335) | 5.447*** | (0.844) | 5.213*** | (0.855) |
| Round-decisions/Individuals/Sessions | 3,098/371/70 | | 3,098/371/70 | | 3,098/371/70 | |
| SD individuals | 2.766 | (0.121) | 2.734 | (0.121) | 2.739 | (0.121) |
| SD session | 1.525 | (0.218) | 1.526 | (0.218) | 1.523 | (0.218) |

**Note.** Standard errors in parentheses. Significance levels: * p<0.05, **p<0.01. ***p<0.001

**Table B1.3.** Multilevel linear models estimating free-riding behavior without and with controls.

| DV: | Free-riding  Model 1 | | Free-riding  Model 2 | | Free-riding  Model 3 | |
| --- | --- | --- | --- | --- | --- | --- |
| *Treatment (ref: Low Trust)* |  |  |  |  |  |  |
| High Trust | -0.004 | (0.308) | -0.048 | (0.280) | 0.145 | (0.384) |
| Age |  |  | 0.011 | (0.012) | 0.011 | (0.012) |
| *Education (ref: Less than Associate degree)* |  |  |  |  |  |  |
| Associate degree or more |  |  | 0.835** | (0.321) | 0.830** | (0.321) |
| *Gender (ref: Male)* |  |  |  |  |  |  |
| Female |  |  | -0.236 | (0.268) | -0.237 | (0.268) |
| *Round (ref: round 1)* |  |  |  |  |  |  |
| Round 2 |  |  | 0.004 | (0.182) | 0.187 | (0.259) |
| Round 3 |  |  | -0.087 | (0.181) | 0.141 | (0.257) |
| Round 4 |  |  | -0.021 | (0.180) | 0.260 | (0.257) |
| Round 5 |  |  | -0.041 | (0.180) | 0.036 | (0.255) |
| Round 6 |  |  | 0.078 | (0.181) | 0.156 | (0.255) |
| Round 7 |  |  | 0.314 | (0.189) | 0.463 | (0.266) |
| Round 8 |  |  | 0.462* | (0.197) | 0.368 | (0.276) |
| Round 9 |  |  | 0.433* | (0.208) | 0.336 | (0.284) |
| Round 10 |  |  | 0.630** | (0.228) | 0.629* | (0.311) |
| High Trust × Round 2 |  |  |  |  | -0.361 | (0.364) |
| High Trust × Round 3 |  |  |  |  | -0.446 | (0.361) |
| High Trust × Round 4 |  |  |  |  | -0.548 | (0.360) |
| High Trust × Round 5 |  |  |  |  | -0.151 | (0.359) |
| High Trust × Round 6 |  |  |  |  | -0.148 | (0.362) |
| High Trust × Round 7 |  |  |  |  | -0.292 | (0.378) |
| High Trust × Round 8 |  |  |  |  | 0.207 | (0.393) |
| High Trust × Round 9 |  |  |  |  | 0.259 | (0.416) |
| High Trust × Round 10 |  |  |  |  | 0.043 | (0.458) |
| *Ethnicity (ref: Other)* |  |  |  |  |  |  |
| White |  |  | 0.141 | (0.380) | 0.133 | (0.380) |
| Conservative Scale |  |  | 0.156 | (0.102) | 0.156 | (0.102) |
| Constant | 2.895*** | (0.219) | 1.252 | (0.686) | 1.153 | (0.699) |
| Round-decisions/Individuals/Sessions | 2,860/369/70 | | 2,860/369/70 | | 2,860/369/70 | |
| SD individuals | 2.376 | (0.109) | 2.343 | (0.109) | 2.345 | (0.109) |
| SD session | 0.673 | (0.224) | 0.420 | (0.314) | 0.409 | (0.321) |

**Note.** Standard errors in parentheses. Significance levels: * p<0.05, **p<0.01. ***p<0.001

**Table B1.4.** Linear probability models estimating achievement of threshold for the public solution without and with controls.

| DV: | Achievement Public Threshold  Model 1 | | Achievement Public Threshold  Model 2 | |
| --- | --- | --- | --- | --- |
| *Treatment (ref: Low Trust)* |  |  |  |  |
| High Trust | 0.343** | (0.107) | 0.430*** | (0.089) |
| Mean Age |  |  | -0.011 | (0.010) |
| % Associate degree or more |  |  | -0.007* | (0.003) |
| % Female |  |  | -0.001 | (0.002) |
| % White |  |  | -0.008* | (0.003) |
| Mean Conservative Scale |  |  | -0.052 | (0.123) |
| Constant | 0.171** | (0.065) | 1.998** | (0.648) |
| Sessions | 70 | | 70 | |
| R^2^ | 0.130 | | 0.281 | |

**Note.** Robust standard errors in parentheses. Significance levels: * p<0.05, **p<0.01. ***p<0.001

**Table B1.5.** Multilevel linear probability models estimating achievement of threshold for the individual solution without and with controls.

| DV: | Achievement Individual Threshold  Model 1 | | Achievement Individual Threshold  Model 2 | |
| --- | --- | --- | --- | --- |
| *Treatment (ref: Low Trust)* |  |  |  |  |
| High Trust | -0.144* | (0.064) | -0.137* | (0.063) |
| Age |  |  | 0.000 | (0.002) |
| *Education (ref: Less than Associate degree)* |  |  |  |  |
| Associate degree or more |  |  | -0.121* | (0.055) |
| *Gender (ref: Male)* |  |  |  |  |
| Female |  |  | 0.014 | (0.045) |
| *Ethnicity (ref: Other)* |  |  |  |  |
| White |  |  | -0.015 | (0.065) |
| Conservative Scale |  |  | -0.068*** | (0.017) |
| Constant | 0.534*** | (0.101) | 0.824*** | (0.145) |
| Individuals/Sessions | 371/70 | | 371/70 | |
| SD sessions | 0. 193 | (0. 032) | 0. 194 | (0. 031) |

**Note.** Standard errors in parentheses. Significance levels: * p<0.05, **p<0.01. ***p<0.001

**Table B1.6.** Linear probability models estimating coordination inefficiency without and with controls.

| DV: | Coordination Inefficiency  Model 1 | | Coordination Inefficiency  Model 2 | |
| --- | --- | --- | --- | --- |
| *Treatment (ref: Low Trust)* |  |  |  |  |
| High Trust | -57.143** | (18.378) | -68.407*** | (16.748) |
| Mean Age |  |  | -0.418 | (1.721) |
| % Associate degree or more |  |  | 1.295* | (0.555) |
| % Female |  |  | -0.044 | (0.359) |
| % White |  |  | 0.826 | (0.591) |
| Mean Conservative Scale |  |  | 4.907 | (24.126) |
| Constant | 273.314*** | (12.472) | 108.335 | (114.092) |
| Sessions | 70 | | 70 | |
| R^2^ | 0.124 | | 0.245 | |

**Note.** Robust standard errors in parentheses. Significance levels: * p<0.05, **p<0.01. ***p<0.001

**Table B1.7.** Linear probability models estimating resources wasted to achieve individual solution without and with controls.

| DV: | Resources Wasted to Achieve  Individual Solution  Model 1 | | Resources Wasted to Achieve  Individual Solution  Model 2 | |
| --- | --- | --- | --- | --- |
| *Treatment (ref: Low Trust)* |  |  |  |  |
| High Trust | -21.257* | (9.490) | -20.395* | (9.306) |
| Mean Age |  |  | -2.514** | (0.845) |
| % Associate degree or more |  |  | 0.009 | (0.294) |
| % Female |  |  | -0.350 | (0.205) |
| % White |  |  | 0.198 | (0.245) |
| Mean Conservative Scale |  |  | 3.168 | (10.073) |
| Constant | 146.486*** | (6.987) | 227.351*** | (51.750) |
| Sessions | 70 | | 70 | |
| R^2^ | 0.069 | | 0.225 | |

**Note.** Robust standard errors in parentheses. Significance levels: * p<0.05, **p<0.01. ***p<0.001

**Table B1.8.** Linear probability models estimating resources wasted to achieve public solution without and with controls.

| DV: | Resources Wasted to Achieve  Public Solution  Model 1 | | Resources Wasted to Achieve  Public Solution  Model 2 | |
| --- | --- | --- | --- | --- |
| *Treatment (ref: Low Trust)* |  |  |  |  |
| High Trust | -35.886* | (16.182) | -48.012** | (14.427) |
| Mean Age |  |  | 2.096 | (1.517) |
| % Associate degree or more |  |  | 1.286* | (0.510) |
| % Female |  |  | 0.305 | (0.367) |
| % White |  |  | 0.628 | (0.530) |
| Mean Conservative Scale |  |  | 1.739 | (18.569) |
| Constant | 126.829*** | (10.684) | -119.016 | (91.487) |
| Sessions | 70 | | 70 | |
| R^2^ | 0.067 | | 0.207 | |

**Note.** Robust standard errors in parentheses. Significance levels: * p<0.05, **p<0.01. ***p<0.001

**Table B1.9.** Logistic models estimating achievement of threshold for the public solution without and with controls.

| DV: | Achievement Public Threshold  Model 1 (OR) | | Achievement Public Threshold  Model 2 (OR) | |
| --- | --- | --- | --- | --- |
| *Treatment (ref: Low Trust)* |  |  |  |  |
| High Trust | 5.118** | (2.895) | 15.663*** | (12.447) |
| Mean Age |  |  | 0.933 | (0.055) |
| % Associate degree or more |  |  | 0.954* | (0.018) |
| % Female |  |  | 0.996 | (0.014) |
| % White |  |  | 0.947** | (0.020) |
| Mean Conservative Scale |  |  | 0.806 | (0.560) |
| Sessions | 70 | | 70 | |

**Note.** OR = Odds Ratio. Robust standard errors in parentheses. Significance levels: * p<0.05, **p<0.01. ***p<0.001

**Table B1.10.** Multilevel logistic models estimating achievement of threshold for the individual solution without and with controls.

| DV: | Achievement Individual Threshold  Model 1 (OR) | | Achievement Individual Threshold  Model 2 (OR) | |
| --- | --- | --- | --- | --- |
| *Treatment (ref: Low Trust)* |  |  |  |  |
| High Trust | 0.438* | (0.162) | 0.438* | (0.172) |
| Age |  |  | 1.002 | (0.013) |
| *Education (ref: Less than Associate degree)* |  |  |  |  |
| Associate degree or more |  |  | 0.517* | (0.166) |
| *Gender (ref: Male)* |  |  |  |  |
| Female |  |  | 1.088 | (0.306) |
| *Ethnicity (ref: Other)* |  |  |  |  |
| White |  |  | 0.918 | (0.372) |
| Conservative Scale |  |  | 0.656*** | (0.075) |
| Individuals/Sessions | 371/70 | | 371/70 | |

**Note.** OR = Odds Ratio. Standard errors in parentheses. Significance levels: * p<0.05, **p<0.01. ***p<0.001

**Table B1.11.** Multilevel linear models estimating public solution investments including inattentive participants, without and with controls.

| DV: | Public Solution  Model 1 | | Public Solution  Model 2 | |
| --- | --- | --- | --- | --- |
| *Treatment (ref: Low Trust)* |  |  |  |  |
| High Trust | 1.213** | (0.422) | 1.244** | (0.415) |
| Age |  |  | 0.001 | (0.011) |
| *Education (ref: Less than Associate degree)* |  |  |  |  |
| Associate degree or more |  |  | -0.188 | (0.294) |
| *Gender (ref: Male)* |  |  |  |  |
| Female |  |  | 0.001 | (0.243) |
| *Round (ref: round 1)* |  |  |  |  |
| Round 2 |  |  | 0.027 | (0.172) |
| Round 3 |  |  | -0.161 | (0.171) |
| Round 4 |  |  | -0.316 | (0.170) |
| Round 5 |  |  | -0.401* | (0.170) |
| Round 6 |  |  | -0.637*** | (0.171) |
| Round 7 |  |  | -0.590*** | (0.173) |
| Round 8 |  |  | -0.670*** | (0.174) |
| Round 9 |  |  | -0.619*** | (0.178) |
| Round 10 |  |  | -0.431* | (0.185) |
| *Ethnicity (ref: Other)* |  |  |  |  |
| White |  |  | -0.480 | (0.346) |
| Conservative Scale |  |  | 0.160 | (0.093) |
| Constant | 2.524*** | (0.299) | 2.915*** | (0.657) |
| Round-decisions/Individuals/Sessions | 3,221/380/70 | | 3,221/380/70 | |
| SD individuals | 2.023 | (0.093) | 2.020 | (0.092) |
| SD session | 1.503 | (0.177) | 1.461 | (0.177) |

**Note.** Participants who did not play or read the instructions for the Trust Game or the Independence Dilemma are included in this analysis. Standard errors in parentheses. Significance levels: * p<0.05, **p<0.01. ***p<0.001

**Table B1.12.** Multilevel linear models estimating individual solution investments including inattentive participants, without and with controls.

| DV: | Individual Solution  Model 1 | | Individual Solution  Model 2 | |
| --- | --- | --- | --- | --- |
| *Treatment (ref: Low Trust)* |  |  |  |  |
| High Trust | -1.198* | (0.469) | -1.220** | (0.468) |
| Age |  |  | -0.007 | (0.015) |
| *Education (ref: Less than Associate degree)* |  |  |  |  |
| Associate degree or more |  |  | -0.289 | (0.378) |
| *Gender (ref: Male)* |  |  |  |  |
| Female |  |  | 0.103 | (0.311) |
| *Round (ref: round 1)* |  |  |  |  |
| Round 2 |  |  | 0.029 | (0.173) |
| Round 3 |  |  | 0.294 | (0.171) |
| Round 4 |  |  | 0.376* | (0.171) |
| Round 5 |  |  | 0.479** | (0.170) |
| Round 6 |  |  | 0.547** | (0.170) |
| Round 7 |  |  | 0.351* | (0.175) |
| Round 8 |  |  | 0.357* | (0.178) |
| Round 9 |  |  | 0.310 | (0.182) |
| Round 10 |  |  | -0.023 | (0.186) |
| *Ethnicity (ref: Other)* |  |  |  |  |
| White |  |  | 0.251 | (0.444) |
| Conservative Scale |  |  | -0.279* | (0.119) |
| Constant | 4.550*** | (0.332) | 5.356*** | (0.822) |
| Round-decisions/Individuals/Sessions | 3,187/382/70 | | 3,187/382/70 | |
| SD individuals | 2.731 | (0.118) | 2.700 | (0.117) |
| SD session | 1.541 | (0.214) | 1.533 | (0.214) |

**Note.** Participants who did not play or read the instructions for the Trust Game or the Independence Dilemma are included in this analysis. Standard errors in parentheses. Significance levels: * p<0.05, **p<0.01. ***p<0.001

**Table B1.13.** Multilevel linear models estimating free-riding behavior including inattentive participants, without and with controls.

| DV: | Free-riding  Model 1 | | Free-riding  Model 2 | |
| --- | --- | --- | --- | --- |
| *Treatment (ref: Low Trust)* |  |  |  |  |
| High Trust | -0.042 | (0.307) | -0.065 | (0.278) |
| Age |  |  | 0.012 | (0.012) |
| *Education (ref: Less than Associate degree)* |  |  |  |  |
| Associate degree or more |  |  | 0.790* | (0.316) |
| *Gender (ref: Male)* |  |  |  |  |
| Female |  |  | -0.231 | (0.262) |
| *Round (ref: round 1)* |  |  |  |  |
| Round 2 |  |  | -0.039 | (0.182) |
| Round 3 |  |  | -0.118 | (0.180) |
| Round 4 |  |  | -0.042 | (0.180) |
| Round 5 |  |  | -0.056 | (0.179) |
| Round 6 |  |  | 0.117 | (0.181) |
| Round 7 |  |  | 0.308 | (0.189) |
| Round 8 |  |  | 0.503* | (0.196) |
| Round 9 |  |  | 0.509* | (0.206) |
| Round 10 |  |  | 0.689** | (0.226) |
| *Ethnicity (ref: Other)* |  |  |  |  |
| White |  |  | 0.177 | (0.368) |
| Conservative Scale |  |  | 0.164 | (0.100) |
| Constant | 2.943*** | (0.217) | 1.228 | (0.670) |
| Round-decisions/Individuals/Sessions | 2,946/380/70 | | 2,946/380/70 | |
| SD individuals | 2.354 | (0.195) | 2.317 | (0.105) |
| SD session | 0.708 | (0.204) | 0.464 | (0.268) |

**Note.** Participants who did not play or read the instructions for the Trust Game or the Independence Dilemma are included in this analysis. Standard errors in parentheses. Significance levels: * p<0.05, **p<0.01. ***p<0.001

**Table B1.14.** Multilevel linear models estimating investments controlling for role in the trust game.

| DV: | Public Solution  Model 1 | | Private Solution  Model 2 | | Free-riding  Model 3 | |
| --- | --- | --- | --- | --- | --- | --- |
| *Treatment (ref: Low Trust)* |  |  |  |  |  |  |
| High Trust | 1.250** | (0.423) | -1.253** | (0.472) | -0.041 | (0.280) |
| Age | 0.004 | (0.012) | -0.008 | (0.015) | 0.011 | (0.012) |
| *Education (ref: Less than Associate degree)* |  |  |  |  |  |  |
| Associate degree or more | -0.175 | (0.298) | -0.345 | (0.385) | 0.832** | (0.321) |
| *Gender (ref: Male)* |  |  |  |  |  |  |
| Female | -0.054 | (0.249) | 0.149 | (0.320) | -0.241 | (0.268) |
| *Round (ref: round 1)* |  |  |  |  |  |  |
| Round 2 | -0.001 | (0.173) | 0.009 | (0.175) | 0.004 | (0.182) |
| Round 3 | -0.186 | (0.172) | 0.285 | (0.173) | -0.087 | (0.181) |
| Round 4 | -0.356* | (0.171) | 0.388* | (0.173) | -0.021 | (0.180) |
| Round 5 | -0.431* | (0.171) | 0.488** | (0.172) | -0.041 | (0.180) |
| Round 6 | -0.626*** | (0.172) | 0.570*** | (0.172) | 0.078 | (0.181) |
| Round 7 | -0.634*** | (0.174) | 0.386* | (0.178) | 0.314 | (0.189) |
| Round 8 | -0.673*** | (0.175) | 0.392* | (0.181) | 0.462* | (0.197) |
| Round 9 | -0.586** | (0.179) | 0.322 | (0.185) | 0.433* | (0.208) |
| Round 10 | -0.415* | (0.186) | 0.006 | (0.189) | 0.629** | (0.228) |
| *Ethnicity (ref: Other)* |  |  |  |  |  |  |
| White | -0.374 | (0.358) | 0.259 | (0.462) | 0.124 | (0.380) |
| Conservative Scale | 0.147 | (0.095) | -0.263* | (0.122) | 0.151 | (0.102) |
| *Role (ref: Truster)* |  |  |  |  |  |  |
| Trustee | 0.156 | (0.228) | -0.422 | (0.297) | 0.237 | (0.259) |
| Constant | 2.694*** | (0.678) | 5.617*** | (0.851) | 1.162 | (0.692) |
| Round-decisions/Individuals/Sessions | 3,135/369/70 | | 3,098/371/70 | | 2,860/369/70 | |
| SD individuals | 2.029 | (0.094) | 2.724 | (0.120) | 2.400 | (0.108) |
| SD session | 1.485 | (0.179) | 1.526 | (0.217) | 0.423 | (0.311) |

**Note.** Standard errors in parentheses. Significance levels: * p<0.05, **p<0.01. ***p<0.001

*B2. Attitudinal outcomes (H4-H5)*

**Table B2.1.** Multilevel linear probability models estimating trusting attitudes without and with controls.

| DV: | Mturk Trust  Model 1 | | Mturk Trust  Model 2 | | Mturk Trustworhiness  Model 1 | | Mturk Trustworhiness  Model 2 | | Social Trust  Model 1 | | Social Trust  Model 2 | |
| --- | --- | --- | --- | --- | --- | --- | --- | --- | --- | --- | --- | --- |
| *Treatment (ref: Low Trust)* |  |  |  |  |  |  |  |  |  |  |  |  |
| High Trust | 0.434** | (0.163) | 0.399* | (0.159) | 0.550*** | (0.160) | 0.505** | (0.158) | 0.240 | (0.134) | 0.187 | (0.128) |
| Age |  |  | -0.008 | (0.006) |  |  | -0.006 | (0.006) |  |  | 0.000 | (0.006) |
| *Education (ref: Less than Associate degree)* |  |  |  |  |  |  |  |  |  |  |  |  |
| Associate degree or more |  |  | 0.438** | (0.159) |  |  | 0.563*** | (0.157) |  |  | 0.500*** | (0.151) |
| *Gender (ref: Male)* |  |  |  |  |  |  |  |  |  |  |  |  |
| Female |  |  | -0.005 | (0.132) |  |  | -0.018 | (0.130) |  |  | -0.103 | (0.125) |
| *Ethnicity (ref: Other)* |  |  |  |  |  |  |  |  |  |  |  |  |
| White |  |  | 0.223 | (0.190) |  |  | 0.237 | (0.187) |  |  | 0.374* | (0.179) |
| Conservative Scale |  |  | 0.230*** | (0.050) |  |  | 0.221*** | (0.049) |  |  | 0.191*** | (0.048) |
| Constant | 3.048*** | (0.116) | 2.145*** | (0.338) | 2.993*** | (0.114) | 1.953*** | (0.333) | 3.199*** | (0.095) | 1.980*** | (0.317) |
| Individuals/Sessions | 371/70 | | 371/70 | | 371/70 | | 371/70 | | 371/70 | | 371/70 | |
| SD sessions | 0.407 | (0.101) | 0. 402 | (0. 095) | 0.387 | (0.104) | 0.407 | (0.094) | 0.170 | (0.174) | 0.146 | (0.012) |

**Note.** Standard errors in parentheses. Significance levels: * p<0.05, **p<0.01. ***p<0.001

**Table B2.2.** Multilevel linear probability models estimating environmental policy support without and with controls.

| DV: | Less Polluting Energy  Model 1 | | Less Polluting Energy  Model 2 | | Increase Fossil Fuel Tax  Model 1 | | Increase Fossil Fuel Tax  Model 2 | |
| --- | --- | --- | --- | --- | --- | --- | --- | --- |
| *Treatment (ref: Low Trust)* |  |  |  |  |  |  |  |  |
| High Trust | 0.209 | (0.107) | 0.190 | (0.105) | 0.061 | (0.140) | 0.045 | (0.139) |
| Age |  |  | -0.006 | (0.005) |  |  | -0.015* | (0.006) |
| *Education (ref: Less than Associate degree)* |  |  |  |  |  |  |  |  |
| Associate degree or more |  |  | 0.184 | (0.127) |  |  | 0.321 | (0.165) |
| *Gender (ref: Male)* |  |  |  |  |  |  |  |  |
| Female |  |  | -0.005 | (0.106) |  |  | 0.122 | (0.137) |
| *Ethnicity (ref: Other)* |  |  |  |  |  |  |  |  |
| White |  |  | 0.040 | (0.151) |  |  | -0.151 | (0.195) |
| Conservative Scale |  |  | -0.195*** | (0.040) |  |  | -0.244*** | (0.052) |
| Constant | 3.923*** | (0.076) | 4.560*** | (0.267) | 3.489*** | (0.100) | 4.610*** | (0.346) |
| Individuals/Sessions | 371/70 | | 371/70 | | 371/70 | | 371/70 | |
| SD sessions | 0.000 | (0.000) | 0. 000 | (0. 134) | 0.000 | (0.013) | 0.146 | (0.208) |

**Note.** Standard errors in parentheses. Significance levels: * p<0.05, **p<0.01. ***p<0.001

**Table B2.3.** Multilevel linear probability models estimating willingness to make personal environmental effort without and with controls.

| DV: | Use less energy  Model 1 | | Use less energy  Model 2 | | Recycle more  Model 1 | | Recycle more  Model 2 | | Save water  Model 1 | | Save water  Model 2 | |
| --- | --- | --- | --- | --- | --- | --- | --- | --- | --- | --- | --- | --- |
| *Treatment (ref: Low Trust)* |  |  |  |  |  |  |  |  |  |  |  |  |
| High Trust | 0.115 | (0.105) | 0.082 | (0.105) | 0.093 | (0.103) | 0.066 | (0.102) | 0.026 | (0.117) | -0.031 | (0.119) |
| Age |  |  | 0.011* | (0.004) |  |  | 0.007 | (0.005) |  |  | 0.011* | (0.005) |
| *Education (ref: Less than Associate degree)* |  |  |  |  |  |  |  |  |  |  |  |  |
| Associate degree or more |  |  | 0.123 | (0.117) |  |  | 0.362** | (0.124) |  |  | 0.427** | (0.137) |
| *Gender (ref: Male)* |  |  |  |  |  |  |  |  |  |  |  |  |
| Female |  |  | 0.163 | (0.097) |  |  | 0.177 | (0.103) |  |  | 0.257* | (0.113) |
| *Ethnicity (ref: Other)* |  |  |  |  |  |  |  |  |  |  |  |  |
| White |  |  | 0.165 | (0.139) |  |  | -0.123 | (0.147) |  |  | 0.125 | (0.162) |
| Conservative Scale |  |  | -0.044 | (0.037) |  |  | 0.004 | (0.039) |  |  | -0.050 | (0.043) |
| Constant | 4.029*** | (0.075) | 3.463*** | (0.247) | 4.055*** | (0.074) | 3.531*** | (0.261) | 3.832*** | (0.084) | 3.037*** | (0.287) |
| Individuals/Sessions | 371/70 | | 371/70 | | 371/70 | | 371/70 | | 371/70 | | 371/70 | |
| SD sessions | 0.184 | (0.093) | 0. 189 | (0. 090) | 0.000 | (0.017) | 0. 000 | (0. 000) | 0.126 | (0.170) | 0. 177 | (0. 122) |

**Note.** Standard errors in parentheses. Significance levels: * p<0.05, **p<0.01. ***p<0.001

**Table B2.4.** Multilevel linear probability models estimating permissiveness towards environmental rule-breaking without and with controls.

| DV: | Acceptable to litter  Model 1 | | Acceptable to litter  Model 2 | | Acceptable not to recycle  Model 1 | | Acceptable not to recycle  Model 2 | |
| --- | --- | --- | --- | --- | --- | --- | --- | --- |
| *Treatment (ref: Low Trust)* |  |  |  |  |  |  |  |  |
| High Trust | 0.151 | (0.204) | 0.108 | (0.153) | 0.088 | (0.152) | 0.093 | (0.124) |
| Age |  |  | -0.021** | (0.006) |  |  | -0.021*** | (0.005) |
| *Education (ref: Less than Associate degree)* |  |  |  |  |  |  |  |  |
| Associate degree or more |  |  | 0.990*** | (0.167) |  |  | 0.347* | (0.143) |
| *Gender (ref: Male)* |  |  |  |  |  |  |  |  |
| Female |  |  | -0.220 | (0.138) |  |  | -0.160 | (0.119) |
| *Ethnicity (ref: Other)* |  |  |  |  |  |  |  |  |
| White |  |  | 0.136 | (0.198) |  |  | -0.006 | (0.170) |
| Conservative Scale |  |  | 0.414*** | (0.053) |  |  | 0.350*** | (0.045) |
| Constant | 2.278*** | (0.145) | 1.051** | (0.352) | 2.851*** | (0.108) | 2.385*** | (0.300) |
| Individuals/Sessions | 371/70 | | 371/70 | | 371/70 | | 371/70 | |
| SD sessions | 0.591 | (0.109) | 0. 310 | (0. 125) | 0. 366 | (0. 100) | 0. 190 | (0. 133) |

**Note.** Standard errors in parentheses. Significance levels: * p<0.05, **p<0.01. ***p<0.001

**Section C – Learning Effects**

In this section, we illustrate how experiences in the Trust Game are affecting behaviors in the Independence Dilemma. Specifically, following the spillover and learning literatures (e.g., Buskens and Raub 2002, Buskens and Raub 2010, Grimm and Mengel 2012, Lo Iacono and Sonmez 2021, Peysakhovich and Rand 2016, Rick and Weber 2010 ), we show how learning to cooperate (or defect) on the basis of what participants observed in the community (i.e., group learning) or experienced individually (i.e., individual learning) influences investments in the Independence Dilemma. Results indicate that group learning is important in increasing investments in the collective solution, but it does not have a significant effect on free-riding or investments in the private solution (see Table C1 and Figure C1). This is in line with results for the one-shot Public Goods Game (Lo Iacono and Sonmez 2021).

Group learning is calculated as the weighted distance between the individual decision at round *j* and the session average decision at round *j* − 1, so that last rounds have more weight than early rounds:


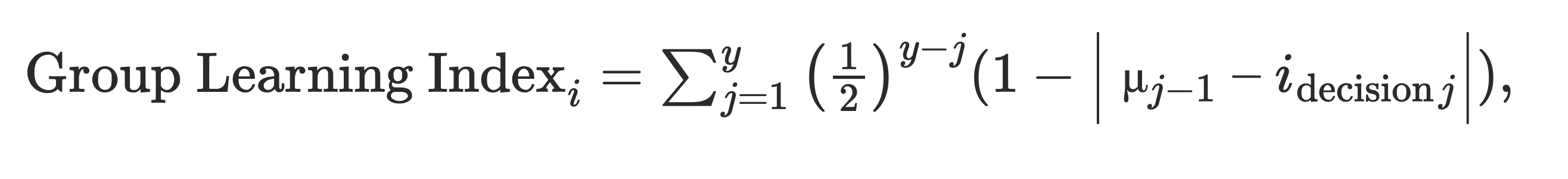


where *y* is the total number of learning occasions (i.e. rounds where player made a decision), *μ* is the mean of the session decisions at round *j* − 1, and *i*_decisionj_ is the decision of player *i* at round *j* (1 = trusting or trustworthy decisions; 0 = distrusting or untrustworthy decisions). Group learning is set to 0 if there are no learning occasions.  The Group Learning Index identifies subjects who progressively adapted to the environment during the iterated TG, assimilating the dominant behavior in the group (see Lo Iacono and Sonmez, 2021).

Individual learning is calculated as follows:


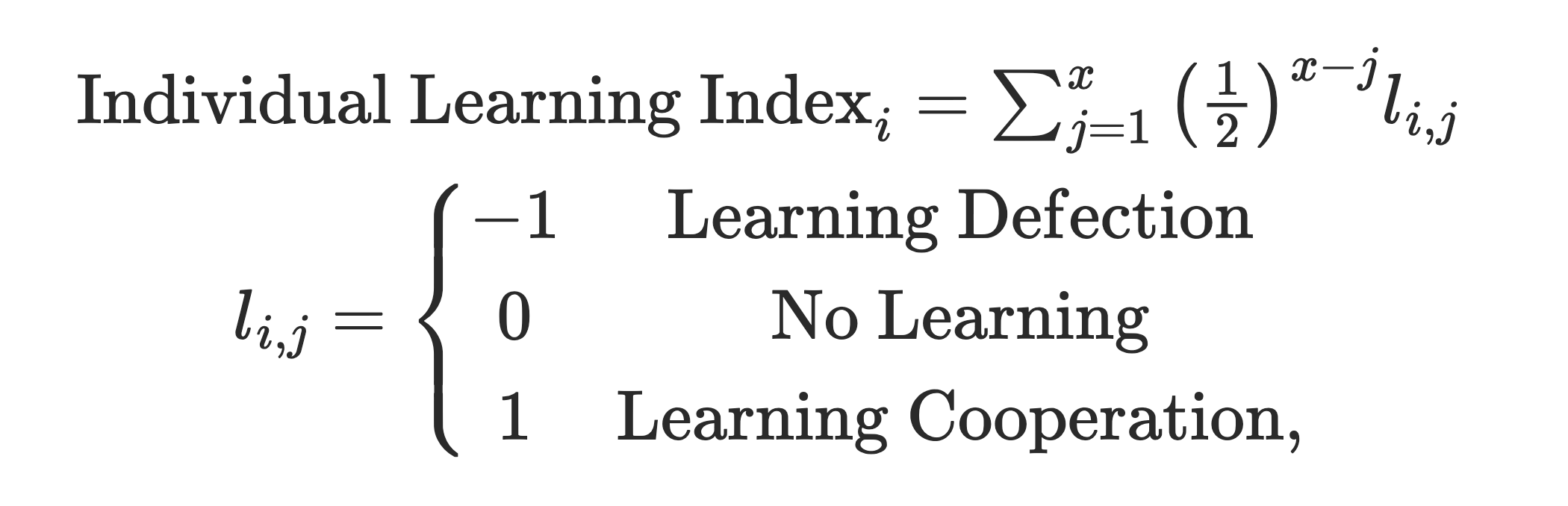


where *x* is the total number of learning occasions (i.e. rounds where both players made a decision). When subject cooperated at round *j* and his/her opponent cooperated at round *j* − 1, learning experience *l_i_*_,_*j* takes a value of 1 to indicate that subject is learning to cooperate. If subject defected at round *j* and his/her opponent defected at round *j* − 1, learning experience *l_i_*_,_*j* takes a value of −1 to indicate that subject is learning to defect. When subjects behaved in a way that is incoherent with their own past experience (i.e. defecting after opponent’s cooperation or cooperating after opponent’s defection), *l_i,j_* takes a value of 0 to indicate that no learning occurred. Individual learning is set to 0 if there are no learning occasions. The Individual Learning index estimates how people learn from their own past experiences in the iterated TG (see Lo Iacono and Sonmez, 2021)

**Table C1.** Multilevel linear models estimating investments and learning effects.

| DV: | Public Solution  Model 1 | | Private Solution  Model 2 | | Free-riding  Model 3 | |
| --- | --- | --- | --- | --- | --- | --- |
| *Treatment (ref: Low Trust)* |  |  |  |  |  |  |
| High Trust | -1.180 | (0.902) | -0.052 | (1.116) | 0.764 | (0.829) |
| Grp Learning | -1.218** | (0.414) | 2.241*** | (0.524) | -1.052* | (0.433) |
| Grp Learning × High Trust | 1.919** | (0.706) | -1.223 | (0.884) | -0.380 | (0.699) |
| Ind Learning | 0.083 | (0.155) | 0.098 | (0.194) | -0.169 | (0.156) |
| Age | 0.002 | (0.011) | -0.006 | (0.014) | 0.010 | (0.012) |
| *Education (ref: Less than Associate degree)* |  |  |  |  |  |  |
| Associate degree or more | -0.067 | (0.300) | -0.214 | (0.379) | 0.491 | (0.323) |
| *Gender (ref: Male)* |  |  |  |  |  |  |
| Female | -0.056 | (0.246) | 0.114 | (0.309) | -0.171 | (0.261) |
| *Round (ref: round 1)* |  |  |  |  |  |  |
| Round 2 | -0.002 | (0.173) | 0.011 | (0.175) | -0.001 | (0.182) |
| Round 3 | -0.185 | (0.172) | 0.286 | (0.173) | -0.091 | (0.181) |
| Round 4 | -0.355* | (0.171) | 0.390* | (0.173) | -0.025 | (0.180) |
| Round 5 | -0.431* | (0.171) | 0.490** | (0.172) | -0.046 | (0.180) |
| Round 6 | -0.624*** | (0.172) | 0.570*** | (0.172) | 0.068 | (0.181) |
| Round 7 | -0.632*** | (0.174) | 0.386* | (0.178) | 0.303 | (0.189) |
| Round 8 | -0.672*** | (0.175) | 0.395* | (0.181) | 0.449* | (0.197) |
| Round 9 | -0.585** | (0.179) | 0.325 | (0.185) | 0.414* | (0.208) |
| Round 10 | -0.415* | (0.186) | 0.008 | (0.189) | 0.613** | (0.228) |
| *Ethnicity (ref: Other)* |  |  |  |  |  |  |
| White | -0.327 | (0.355) | 0.203 | (0.447) | 0.107 | (0.370) |
| Conservative Scale | 0.110 | (0.094) | -0.215 | (0.118) | 0.113 | (0.100) |
| *Role (ref: Truster)* |  |  |  |  |  |  |
| Trustee | 0.037 | (0.232) | -0.148 | (0.293) | 0.073 | (0.257) |
| Constant | 4.271*** | (0.857) | 2.631* | (1.075) | 2.798** | (0.883) |
| Round-decisions/Individuals/Sessions | 3,135/369/70 | | 3,098/371/70 | | 2,860/369/70 | |
| SD individuals | 2.010 | (0.095) | 2.610 | (0.117) | 2.263 | (0.105) |
| SD session | 1.388 | (0.181) | 1.620 | (0.225) | 0.434 | (0.288) |

**Note.** Standard errors in parentheses. Significance levels: * p<0.05, **p<0.01. ***p<0.001

**Figure C1.** Investments in the collective solution by treatment and group learning.

**
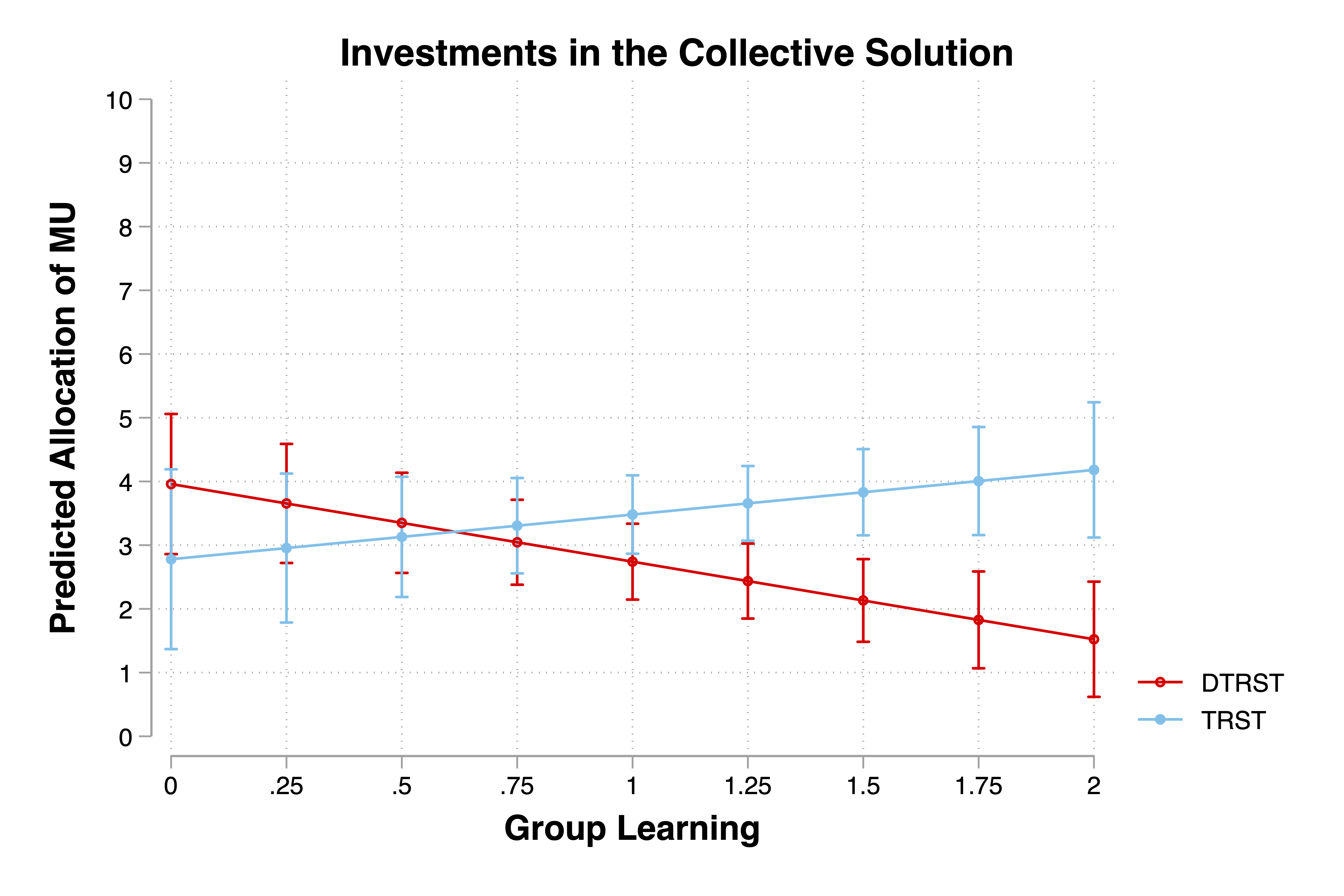
**

**Note.** Predicted values for investments in the collective solution by treatment and group learning with 95% CIs based on a multilevel linear models with round-decisions nested in individuals nested in groups (N round-decisions/individuals/ groups = 3,135/369/70) and controls for age, education, gender, ethnicity, political orientation, role, rounds, and individual learning (Model 1, Table C1).

**Reference List**

Buskens, Vincent, and Werner Raub. 2002. "Embedded trust: Control and learning." Pp. 167-202 in Advances in group processes: Emerald Group Publishing Limited.

Buskens, Vincent, Werner Raub, and Joris van der Veer. 2010. "Trust in triads: An experimental study." Social Networks 32(4):301-12.

Chen, Daniel L, Martin Schonger, and Chris Wickens. 2016. "oTree—An open-source platform for laboratory, online, and field experiments." Journal of Behavioral and Experimental Finance 9:88-97.

Cheung, Janelle H, Deanna K Burns, Robert R Sinclair, and Michael Sliter. 2017. "Amazon Mechanical Turk in organizational psychology: An evaluation and practical recommendations." Journal of Business and Psychology 32(4):347-61.

Ermisch, John, Diego Gambetta, Heather Laurie, Thomas Siedler, and SC Noah Uhrig. 2009. "Measuring people's trust." Journal of the Royal Statistical Society: Series A (Statistics in Society) 172(4):749-69.

Grimm, V., & Mengel, F. (2012). An experiment on learning in a multiple games environment. Journal of Economic Theory, 147(6), 2220-2259.

Gross, Jörg, and Carsten KW De Dreu. 2019. "Individual solutions to shared problems create a modern tragedy of the commons." Science advances 5(4):eaau7296.

Lo Iacono, S., & Sonmez, B. (2021). The effect of trusting and trustworthy environments on the provision of public goods. European Sociological Review, 37(1), 155-168.

Moss, Aaron J, Cheskie Rosenzweig, Jonathan Robinson, and Leib Litman. 2020. "Demographic stability on Mechanical Turk despite COVID-19." Trends in cognitive sciences 24(9):678-80.

Peer, Eyal, Joachim Vosgerau, and Alessandro Acquisti. 2014. "Reputation as a sufficient condition for data quality on Amazon Mechanical Turk." Behavior research methods 46(4):1023-31.

Peysakhovich, A., & Rand, D. G. (2016). Habits of virtue: Creating norms of cooperation and defection in the laboratory. Management Science, 62(3), 631-647.

Rick, S., & Weber, R. A. (2010). Meaningful learning and transfer of learning in games played repeatedly without feedback. Games and Economic Behavior, 68(2), 716-730.

Robinson, Jonathan, Cheskie Rosenzweig, Aaron J Moss, and Leib Litman. 2019. "Tapped out or barely tapped? Recommendations for how to harness the vast and largely unused potential of the Mechanical Turk participant pool." PloS one 14(12):e0226394.
